# Association of Lower-Limb Asymmetry with Falls, Physical Performance and Frailty in Older Adults: A Systematic Review

**DOI:** 10.3390/jcm15208198

**Published:** 2026-10-21

**Authors:** Krzysztof Kasicki, Kacper Szymeczko

**Affiliations:** 1 Department of Sport Theory and Motor Skills, Institute of Sports Sciences, University of Physical Culture in Kraków, 31-571 Kraków, Poland; 2 Faculty of Health Sciences, Andrzej Frycz Modrzewski Krakow University, 30-705 Kraków, Poland; kszymeczko@uafm.edu.pl

**Keywords:** interlimb asymmetry, fall risk, strength, gait, frailty, older adults, functional tests, physical fitness

## Abstract

**Background/Objectives:** Whether lower-limb interlimb asymmetry is associated with adverse outcomes in older adults independently of bilateral strength, power or muscle mass remains unclear. This review synthesized evidence linking lower-limb asymmetry with falls, physical performance and frailty in adults aged ≥ 60 years from populations not defined by a unilateral neurological or musculoskeletal condition. **Methods:** Following PRISMA 2020, PubMed, Scopus, Web of Science and EBSCOhost were searched on 2 August 2026 for observational studies quantifying lower-limb asymmetry. Risk of bias was assessed with the Newcastle–Ottawa Scale and AXIS. Because exposure definitions and effect measures were incompatible, meta-analysis was not feasible; a narrative synthesis based on the direction of effect was conducted according to SWiM, and certainty of evidence was rated with GRADE. **Results:** Twenty-six studies (10,167 participants; 16 cross-sectional, 6 case–control, 4 prospective cohorts) were included. Findings for strength and power asymmetry were inconsistent, and the largest prospective cohort (*n* = 5347) found no association with recurrent falls. Greater muscle mass asymmetry was associated with slower gait speed in three cross-sectional studies. Selected gait and loading asymmetry measures were associated with subsequent falls and incident frailty in two small cohorts. Positive findings for falls came mainly from case–control studies with retrospective, self-reported fall ascertainment, whereas the studies at the lowest risk of bias did not confirm them. Certainty of evidence was very low for all eight exposure–outcome pairs, including muscle mass asymmetry and gait speed. **Conclusions:** Lower-limb asymmetry is not a homogeneous construct, and its associations, which depend on the measurement domain and outcome, should be regarded as exploratory. Current evidence does not establish the independent predictive value of asymmetry and supports neither a universal asymmetry threshold nor its use as a standalone fall-risk marker; it should be interpreted alongside bilateral muscle capacity and task demands.

## 1. Introduction

Falls are one of the major health problems in older adults [1]. According to Global Burden of Disease data, the age-standardized incidence of falls in the general population in 2017 was 2238 per 100,000 people [2]. In older adults, falls lead to fractures, hospitalization, loss of independence and premature death [1]. International guidelines recommend a multifactorial fall risk assessment that includes, among other components, an assessment of physical performance and mobility [1]. The tools used for this purpose, such as the Short Physical Performance Battery, are based on aggregate indicators of lower-limb function. Their scores are associated with disability and predict institutionalization and mortality [3].

Muscle mass and strength decline with age, as described by the concept of sarcopenia [4]. Lower muscle power has also been reported in older adults with a history of falls [5,6]. Moreover, reduced strength and slower gait are among the criteria for the frailty phenotype, which increases the risk of falls, worsening mobility and loss of independence [7].

Measures of muscle mass, strength and power are most often analyzed as absolute or aggregate values, without considering their distribution between the limbs [4]. Two individuals with the same summary score may differ in the degree of imbalance between the right and left sides. This matters because gait requires alternating weight transfer, and maintaining balance requires the coordinated action of both limbs.

A certain degree of asymmetry is physiologically present in the gait of healthy individuals [8]. With age, bilateral coordination of gait deteriorates; it constitutes a distinct dimension of locomotor control that is only partly explained by gait asymmetry, speed and variability [9]. Much of the methodology for the quantitative assessment of interlimb asymmetry was developed in sports science. Numerous indices and percentage thresholds have been proposed, most commonly 10–15% [10,11]. These indices have computational limitations arising from the choice of the reference limb and the normalization method [11], and the interpretation of the cut-off values used has not been unequivocally established [10]. Moreover, they were developed in young, trained individuals, in whom both the causes of asymmetry and the outcomes analyzed differ from those observed in the geriatric population. In older adults, the same asymmetry corresponds to a smaller between-limb difference, is superimposed on the age-related decline in capacity and may arise from causes other than sport-specific loading. Whether thresholds derived from athletes identify functionally relevant asymmetry in older adults therefore remains an open question that this review may potentially help to answer.

Findings concerning the upper limb suggest that asymmetry may provide additional prognostic information. In a sample of 18,468 Americans aged 50 years and older, the co-occurrence of handgrip strength asymmetry and weakness was associated with future disability in basic activities of daily living. The authors concluded that the assessment of asymmetry may increase the predictive value of dynamometry [12]. However, this conclusion cannot be directly generalized to the lower limb, which has weight-bearing and locomotor functions and whose asymmetry is assessed across many distinct dimensions.

The results of previous studies in older adults are inconclusive. Some authors found greater strength and power asymmetry in fallers or in individuals with poorer performance [5,13]. Others did not confirm this relationship or showed only a weak association [6,14,15]. In the same studies, the outcomes were clearly associated with the absolute level of strength and power [6,14,15]. Muscle mass asymmetry [16,17] and gait, loading and coordination parameters [18,19,20,21] have been studied separately. The relationship between these indices has not been established.

The associations observed in smaller samples were not straightforwardly confirmed in the two largest studies. These studies concerned lower-limb power asymmetry [22] and foot force asymmetry [21].

The upper and lower limbs were assessed simultaneously in two studies. In these studies, upper-limb asymmetry was associated with recurrent falls [22] and with the development of mobility limitation [19], respectively, whereas lower-limb asymmetry showed no such association in either of them. Because both limbs were assessed using the same task and measure in one of these studies [19], the difference cannot be attributed solely to the method of measurement.

These discrepancies are difficult to resolve on the basis of individual studies. They may partly result from the heterogeneity of the exposure itself. Asymmetry is defined and calculated in different ways, depending on the measurement dimension and the formula applied [11]. The methods of categorizing asymmetry, including the threshold values used, also differ, and study results are presented using non-comparable scales and effect estimates.

It also remains unknown whether asymmetry provides prognostic information beyond the bilateral values of strength, power or muscle mass, given that it is itself calculated from them [14,15]. The values of parameters such as strength, power or muscle mass and the degree of their interlimb asymmetry are related but distinct constructs. Individuals with a similar asymmetry index may differ in the absolute values of these parameters, just as a similar level of strength, power or muscle mass does not necessarily imply a comparable degree of asymmetry. Demonstrating that asymmetry provides information beyond the absolute values of the analysed parameters requires confirmation of its association with the outcome after accounting for the values obtained for both limbs. When assessing prognostic value, a prospective design is necessary, in which the measurement of asymmetry precedes the occurrence of the outcome. Cross-sectional and retrospective studies make it possible to identify associations between variables, but they do not provide a sufficient basis for confirming the prospective predictive value of asymmetry.

To our knowledge, no systematic review has yet been published that compiles this evidence separately for individual measurement domains and types of clinical outcomes and assesses its certainty. Because asymmetry indices derived from different measurement dimensions capture different constructs and are not interchangeable, we prespecified five measurement domains, described below in Section 2, to avoid combining non-equivalent exposures in a single synthesis.

### Objectives

The aim of this systematic review was to synthesize the evidence on the association between quantified lower-limb interlimb asymmetry and falls, physical performance and frailty in individuals aged 60 years and older. This review focuses on interlimb asymmetry in the general older population, that is, in individuals who were not enrolled in studies on the basis of a diagnosis of a specific neurological or musculoskeletal condition.

The specific objectives of this study were as follows:

(A1) To characterize the methods of quantifying lower-limb asymmetry used in studies of older populations;
(A2) To compile the direction and magnitude of the observed associations separately for each prespecified exposure domain and for each outcome type;
(A3) To determine whether the association between asymmetry and the outcome persists after accounting for muscle strength, power or mass;
(A4) To formally assess the certainty of evidence using the GRADE approach separately for each exposure domain–outcome pair.

## 2. Materials and Methods

The review was prepared in accordance with the PRISMA 2020 guidelines [23]. The completed PRISMA 2020 checklist is provided in the Supplementary Materials (Table S1). Owing to the anticipated heterogeneity of exposure measures, the synthesis was planned as a structured narrative synthesis, reported in accordance with the Synthesis Without Meta-analysis (SWiM) guideline [24], and the certainty of evidence was assessed using the GRADE approach [25]. The review protocol was registered in PROSPERO under registration number CRD420261512036. During the preparation of this manuscript, the authors used the large language model Gemini 3.1 Pro (Google LLC, Mountain View, CA, USA) for translation and grammatical correction.

### 2.1. Eligibility Criteria

The review question was formulated according to the PECOS framework (population, exposure, comparator, outcomes, study design). Given the prognostic–descriptive nature of the review, the comparator was defined as comparative contrasts specified a priori and analyzed within the collected body of evidence.

The exposure was divided a priori into five measurement domains: (A) muscle strength and power; (B) limb functional capacity; (C) muscle mass, composition and quality; (D) spatiotemporal gait parameters; (E) kinetics, kinematics and postural control. This division determined both the structure of data extraction and the structure of the synthesis.

Studies meeting all of the following criteria were eligible for the review:

I1. Population: Individuals aged 60 years and older, living in the community, in care facilities or receiving long-term care. Studies covering a wider age range were eligible only if an analysis restricted to participants aged 60 years and older was presented.
I2. Exposure: A quantitative measure of lower-limb interlimb asymmetry, expressed as a continuous or categorized variable, assigned to at least one of the five prespecified measurement domains.
I3. Direction of the relationship: Asymmetry analyzed as an exposure preceding or co-occurring with the outcome.
I4. Outcome: at least one of three prespecified outcomes: Falls; physical performance or mobility; and frailty.
I5. Reporting: Presentation of a quantitative measure of the association between exposure and outcome or of a between-group comparison allowing the direction of this association to be determined.
I6. Study design: Observational study—cross-sectional, case–control or cohort.

Studies were excluded from the review on the basis of the following criteria:

E1. Reversed direction of the analyzed relationship.
E2. Methodological papers not reporting any clinical outcome.
E3. Exposure other than lower-limb asymmetry.
E4. Outcome reported only for the side affected by pathology.
E5. Exposure defined as asymmetry of passive range of motion.
E6. Sample not restricted to individuals aged 60 years and older.
E7. Population defined by a unilateral pathology, e.g., stroke.
E8. Publication type: narrative and systematic reviews, meta-analyses, bibliometric studies, editorials, letters to the editor, commentaries, notes, conference abstracts and book chapters.

Populations defined by a unilateral pathology (E7) were excluded because, in such groups, asymmetry is a direct manifestation of the lesion, and its magnitude typically far exceeds that observed in the general older population. Combining these populations with the general older population would conflate two different exposures. For the same reason, results reported only for the side affected by pathology were excluded (E4).

Criterion I3 admitted asymmetry measured either before the occurrence of the outcome or at the same time. The body of evidence comprises both prognostic and associative findings. To avoid conflating association with prediction, these were distinguished throughout the synthesis: inferences about the predictive value of asymmetry were restricted to prospective cohorts, whereas the findings of cross-sectional and case–control studies were interpreted as evidence of association.

No restrictions on the date of publication were applied. Publications were eligible without language restrictions. Neither a minimum sample size nor statistical significance of the reported associations was required.

### 2.2. Search Strategy and Information Sources

The search was conducted on 2 August 2026 in four sources: PubMed, Scopus, Web of Science and the databases available on the EBSCOhost platform. The search covered the period from the inception of each source to the date of the search. Clinical trial registers and grey literature sources were not searched; this decision was made a priori and was taken into account in the assessment of the risk of reporting bias. The search was not repeated before submission of the manuscript; consequently, studies published after 2 August 2026 could not be included.

The search strategies were developed by the first author (K.K.), and their completeness and correctness were verified independently by the second author (K.S.). The strategies were built from four conceptual blocks covering, respectively: older age; asymmetry or symmetry and related concepts; the lower limbs, gait and the musculoskeletal structures of the lower limb; and falls, balance, postural control, gait speed, physical performance, mobility, sarcopenia and frailty. Synonyms within a block were combined with the OR operator, and the blocks were combined using the AND operator. Truncation and quotation marks for multi-word phrases were used. The full search strings for each source are presented in Appendix A.

### 2.3. Study Selection Process

Records retrieved from all sources were exported and merged into a single set, and duplicates were removed in Microsoft Excel (Microsoft Corporation, Redmond, WA, USA) in four consecutive steps:

1. Matching of identical DOIs;
2. Matching of identical PMIDs;
3. Matching of identical titles with a difference in the year of publication not exceeding one year;
4. Approximate matching of titles, subsequently verified manually.

The numbers of records removed at each deduplication step are given in Section 3.1.

The titles and abstracts of all records remaining after deduplication were screened independently and in parallel by two reviewers (K.K. and K.S.), blinded to each other’s decisions. Before screening began, a calibration exercise was performed on a random sample of records in order to harmonize the interpretation of the eligibility criteria. The full texts were then assessed independently by the same two reviewers, who assigned each excluded report a single primary exclusion criterion according to the E1–E8 hierarchy. Disagreements were resolved through discussion and, if no agreement was reached, the decision was made by a third assessor (T.A.), whose ruling was binding. Inter-rater agreement between the two reviewers was assessed using Cohen’s kappa coefficient (κ = 0.62 at the title–abstract screening stage and κ = 0.74 at the full-text screening stage).

The unit of analysis was the study. The included studies were checked for multiple reports from the same sample. Two reports from the same research team based on potentially overlapping samples [19,26] were treated as separate studies because they differed in design and sample size; this potential overlap was noted when interpreting the consistency of findings within the relevant domain. To limit the risk of over-weighting the same data, the two reports were not treated as independent sources confirming the findings. Omitting either of them would not have affected the final certainty-of-evidence rating for the exposure–outcome pair to which both reports contributed data. The selection process is presented in the PRISMA 2020 flow diagram in Section 3.1.

### 2.4. Data Extraction

Data were extracted by one reviewer (K.K.) using a standardized, previously prepared data extraction form, and the completeness and correctness of all items were verified in full by a second reviewer (K.S.). Disagreements were resolved through discussion and, if no agreement was reached, by a third assessor not involved in the formal preparation of the review (T.A.). The form was piloted on three studies with different designs, after which the definitions of the fields concerning exposure categorization and the extent of adjustment for confounders were refined.

From each included study, the following information was obtained: bibliographic details and the country in which the study was conducted, study design and, for longitudinal studies, the duration and method of follow-up, sample size, age and sex of participants, recruitment setting, and the inclusion and exclusion criteria applied in the primary study. The exposure domain, a detailed definition of asymmetry together with the formula used to calculate it, the measurement equipment used, the method of categorization and the reported measurement reliability indices were also extracted. For each outcome, its definition and method of ascertainment, the effect estimate together with its measure of precision, and the variables included in the multivariable models were recorded. In addition, the use of a correction for multiple testing, funding sources and declared conflicts of interest were noted.

If a study reported more than one asymmetry index or more than one outcome, all findings within the scope of the review were extracted in order to avoid selective inclusion of results by the review team. If a given association was presented both in an unadjusted analysis and in an adjusted model, priority in the synthesis was given to the estimate from the model with the most complete adjustment for confounders, while retaining information on the result of the unadjusted analysis. For results presented only graphically, numerical values were not extracted from the graphs, and the finding was classified solely in terms of the direction of the effect.

### 2.5. Risk of Bias Assessment in Included Studies

The risk of bias was assessed at the level of the individual study using two tools selected according to the study design. Prospective cohorts and case–control studies were assessed with the Newcastle–Ottawa Scale (NOS) [27], and cross-sectional studies with the Appraisal Tool for Cross-Sectional Studies (AXIS) [28]. The use of two instruments resulted from the fact that the NOS domains concerning the selection of the non-exposed group and the temporal sequence of exposure and outcome are not applicable to cross-sectional studies; imposing a single tool on the whole set would lead to assessments that appear uniform but are substantively incomparable.

One modification, established a priori, was introduced to the NOS. The second item of the comparability domain, which the tool authors leave for the review team to define, was assigned to adjustment for muscle strength, power or mass. In this review, this variable is a key confounder: asymmetry is a quantity derived from the bilateral values, and the question of its incremental value is, by definition, a question about an association that persists after these values have been accounted for.

Each study was assessed independently by two reviewers (K.K. and K.S.), and disagreements were resolved by a third assessor (T.A.). The total score was categorized as low risk of bias (7–9), moderate (5–6) or high (≤4). In AXIS, each of the twenty items was assigned a value of 1 (criterion met), 0.5 (partially met or insufficiently reported) or 0 (criterion not met); items 13 and 19, which are phrased negatively in the original tool, were reversed so that a higher value indicated a more favorable rating for all items. The sum of points was categorized as low risk of bias (≥17/20), moderate (12–16.5) or high (<12). The categorization thresholds were set before the assessments began, because neither tool defines them itself. The NOS thresholds are consistent with the convention used in systematic reviews, whereas the AXIS thresholds were set by analogy, that is, approximately 85% and 60% of the maximum score for low and moderate risk, respectively. Because these categories are not intrinsic to either instrument, they were used descriptively only. Total scores obtained with the NOS and AXIS are not directly comparable.

The risk of bias assessments were not used as an exclusion criterion. They were used twice: when weighting individual findings in the narrative synthesis and when considering downgrading for risk of bias within GRADE.

### 2.6. Effect Measures and Standardized Synthesis Metric

The prespecified effect measure was the odds ratio or relative risk for dichotomous outcomes and the standardized mean difference for continuous outcomes. However, at the data extraction stage, it became clear that the condition necessary for deriving a common effect measure was not met: the included studies used definitions of exposure that could not be converted to a common metric and estimators reported on incompatible scales. Transforming these quantities to a single scale would require assumptions about the distribution of the exposure and the linearity of the associations that could not be verified using the available data and that would predetermine the result of the synthesis.

For this reason, meta-analysis was abandoned and, in accordance with the SWiM guidelines [24], the direction of effect was adopted as the standardized metric. The direction was classified into one of four categories: positive, when greater asymmetry was associated with a worse outcome; no association; measure-dependent, when the presence or direction of the association differed between outcome measures or exposure indices within the same study; and inverse, when greater asymmetry was associated with a better outcome. Classification was based on the point estimate and its confidence interval or on a between-group comparison. For each finding, the effect estimate and its measure of precision were extracted where they were reported in the primary study; they are presented in Section 3, together with the reason when no estimate of precision was available. No formal vote counting or statistical test of the distribution of directions was performed, because a method of this type ignores effect size and sample size and, in a set with such varied sample sizes, would lead to misleading conclusions. Of the other methods listed in the SWiM guidelines as alternatives to meta-analysis [24], summarizing effect estimates was not possible because the estimates were reported on incompatible scales, and combining *p*-values was not appropriate because exact *p*-values for a common one-sided hypothesis were rarely reported. The direction of effect was the only metric that could be determined for all 26 studies. The limitation that the direction of effect does not convey precision or clinical relevance was mitigated by interpreting each finding together with its estimate and other variables, such as sample size.

### 2.7. Grouping of Studies for Synthesis

Studies were grouped for synthesis by exposure domain according to the classification adopted a priori, and the resulting division was cross-tabulated by outcome domain. The criterion for assignment was the construct measured by the asymmetry index.

After data extraction had been completed, two sets of domains were merged. Domains A (muscle strength and power) and B (limb functional capacity) were merged because eight of the twelve studies in this group measured both constructs in the same participants and reported them within a single model, which made their separation artificial. Domains D (spatiotemporal gait parameters) and E (kinetics, kinematics and postural control) were combined for the same reason: loading asymmetries and kinetic variables were reported jointly, and six studies contributed data to both domains. Both modifications were introduced before the synthesis began and before any summary of the direction of effect was prepared.

For studies providing data relating to more than one domain, the full results were presented in the domain corresponding to the primary exposure, i.e., the one directly related to the main research question of that study, whereas appropriate cross-references were included in the secondary domains. This approach made it possible to avoid counting the same study more than once in the summary tables. As a result, the numbers of studies reported in the subsections on individual domains refer only to their primary classification, and the sum of assignments across all domains corresponds to the total number of studies included in the review.

### 2.8. Synthesis Methods

The narrative synthesis was conducted according to a predefined structure. Within each exposure domain, studies were ordered first by study design and then, within individual categories, by decreasing sample size. This scheme corresponded to the a priori criteria for prioritizing the evidence. Greater interpretive weight was assigned to studies allowing assessment of the temporal sequence, to studies with larger samples and to analyses accounting for the level of muscle strength, power or mass. When interpreting the results, the risk of bias assessment conducted according to the procedure described in Section 2.5 was additionally taken into account. Findings from prospective cohorts were interpreted as evidence on prediction, whereas findings from cross-sectional and case–control studies were interpreted as evidence of association.

Conclusions were not formulated based on the number of results reaching statistical significance, and significance alone was not a criterion for including a finding in the synthesis. In domains in which the direction of effect was heterogeneous, the studies reporting divergent findings were identified, and these findings were related to the sample size and the risk of bias assessment of these studies. Summaries of the direction of effect across exposure domains and across outcome domains are presented in tabular form in Section 3.5 and Section 3.6. These tables serve to organize the evidence and do not constitute a quantitative synthesis; the numbers of studies in individual categories describe how the findings are distributed and are not a measure of the strength or certainty of evidence; they should not be read as vote counting.

### 2.9. Investigation of Heterogeneity of Findings

Owing to the lack of a common effect measure, statistical heterogeneity indices were not calculated. Potential sources of variability in the results were analyzed in a structured manner by comparing the direction of the observed effects across predefined study characteristics. These included the exposure and outcome domain, study design, sample size, the method of outcome ascertainment (distinguishing between prospective monitoring of falls and their retrospective ascertainment on the basis of self-report), the inclusion or absence of adjustment for neuromuscular capacity, the method of exposure modeling and the participant recruitment setting.

In addition, the reported indices of exposure measurement reliability were noted, because non-differential misclassification of exposure shifts estimates toward the null and constitutes an alternative explanation for negative findings. The analysis of threshold effects and non-monotonic associations presented in Section 3.7 was conducted after the data had been examined; it is exploratory and hypothesis-generating in nature, as indicated in the text, and was not part of the registered analysis plan. Owing to the small number of studies in each potential subgroup, formal subgroup analyses by variables such as sex or baseline functional status were not feasible; these characteristics were examined descriptively.

### 2.10. Assessment of Risk of Bias Due to Missing Results

An assessment of publication bias using a funnel plot and an asymmetry test was planned; this assessment proved infeasible given the lack of a common and comparable effect measure. Instead, the risk of bias due to missing results was assessed qualitatively. The distribution of the direction of findings was compiled and compared with the sample size and the risk of bias assessment of individual studies. Next, for each study, the number of asymmetry indices analyzed, the number of results reported as significant, and whether a correction for multiple testing was used were recorded. The scope of analyses announced in the methods section of the primary study was compared with the scope of analyses actually reported. The lack of registration of the protocols of primary studies was not in itself considered evidence of selective reporting, but it was treated as a limitation preventing this source of bias from being reliably ruled out.

### 2.11. Assessment of Certainty of Evidence

The certainty of evidence was assessed using the GRADE approach [25] separately for each exposure domain–outcome pair. The assessment of each pair started at a low level of certainty appropriate for bodies of evidence consisting solely of observational studies, and downgrading was then considered in five domains: risk of bias, inconsistency, indirectness, imprecision and publication bias. Downgrading by one level was labeled “serious” and by two levels “very serious”. Three criteria for upgrading were also considered: a large effect size, the presence of a dose–response gradient and a situation in which the confounders accounted for would act against the observed effect.

The GRADE assessment was performed independently by two reviewers (K.K. and K.S.), and disagreements were resolved through discussion with the participation of a third assessor who had the status of an independent reviewer (T.A.). Owing to the lack of a common effect measure, imprecision was assessed on the basis of the total number of participants and the width of the confidence intervals reported in individual studies. Inconsistency of results was judged on the basis of the variation in the directions of the observed effects. Limitations arising from the observational nature of the studies were accounted for in the initial certainty-of-evidence rating and in the risk of bias domain. They did not constitute an additional basis for downgrading in the indirectness domain, to avoid counting the same methodological limitations more than once. Indirectness was judged in terms of the correspondence of the studied populations, the methods of exposure measurement and the analysed outcomes to the research question, taking into account, among other things, the use of surrogate indicators of fall risk.

### 2.12. Deviations from the Registered Protocol

The systematic review was conducted in accordance with the protocol registered in PROSPERO (CRD420261512036), taking into account the deviations described below. The protocol specified a priori the odds ratio and the standardized mean difference as the planned effect measures, providing for a meta-analysis where appropriate data were available, or a structured narrative synthesis where a quantitative synthesis would not be justified. Because no common effect measure could be applied, the meta-analysis and the planned funnel plot-based assessment of publication bias were not performed. Instead, a synthesis based on the direction of effect was used, in accordance with the SWiM guidelines [24]. Domains A and B and domains D and E were merged after data extraction for the reasons given in Section 2.7. Both of these decisions were made before any summary of the direction of effect was prepared. The analysis of threshold and non-monotonic associations was not prespecified; it was conducted after the data had been examined and is reported as a post hoc, hypothesis-generating analysis. No other deviations from the registered protocol occurred.

## 3. Results

### 3.1. Study Selection

The searches conducted on 2 August 2026 returned 7822 records: Web of Science Core Collection (*n* = 3431), PubMed (*n* = 2490), Scopus (*n* = 1366) and EBSCOhost (*n* = 535). No records were identified from study registers or grey literature. Deduplication was performed in four consecutive steps and removed 2916 records: 2489 on the basis of identical DOIs, 14 on the basis of identical PMIDs, 410 on the basis of the same title with a difference in the year of publication not exceeding one year, and 3 on the basis of approximate title matching. Deduplication was performed in Microsoft Excel. A total of 4906 records remained.

The titles and abstracts of all 4906 records were assessed, and 4372 were excluded because of failure to meet the population age criterion (*n* = 1723), a population defined by a unilateral pathology or a study involving athletes, children or animals (*n* = 1631), and the absence of a quantitative measure of lower-limb asymmetry (*n* = 1018).

**Figure 1:**
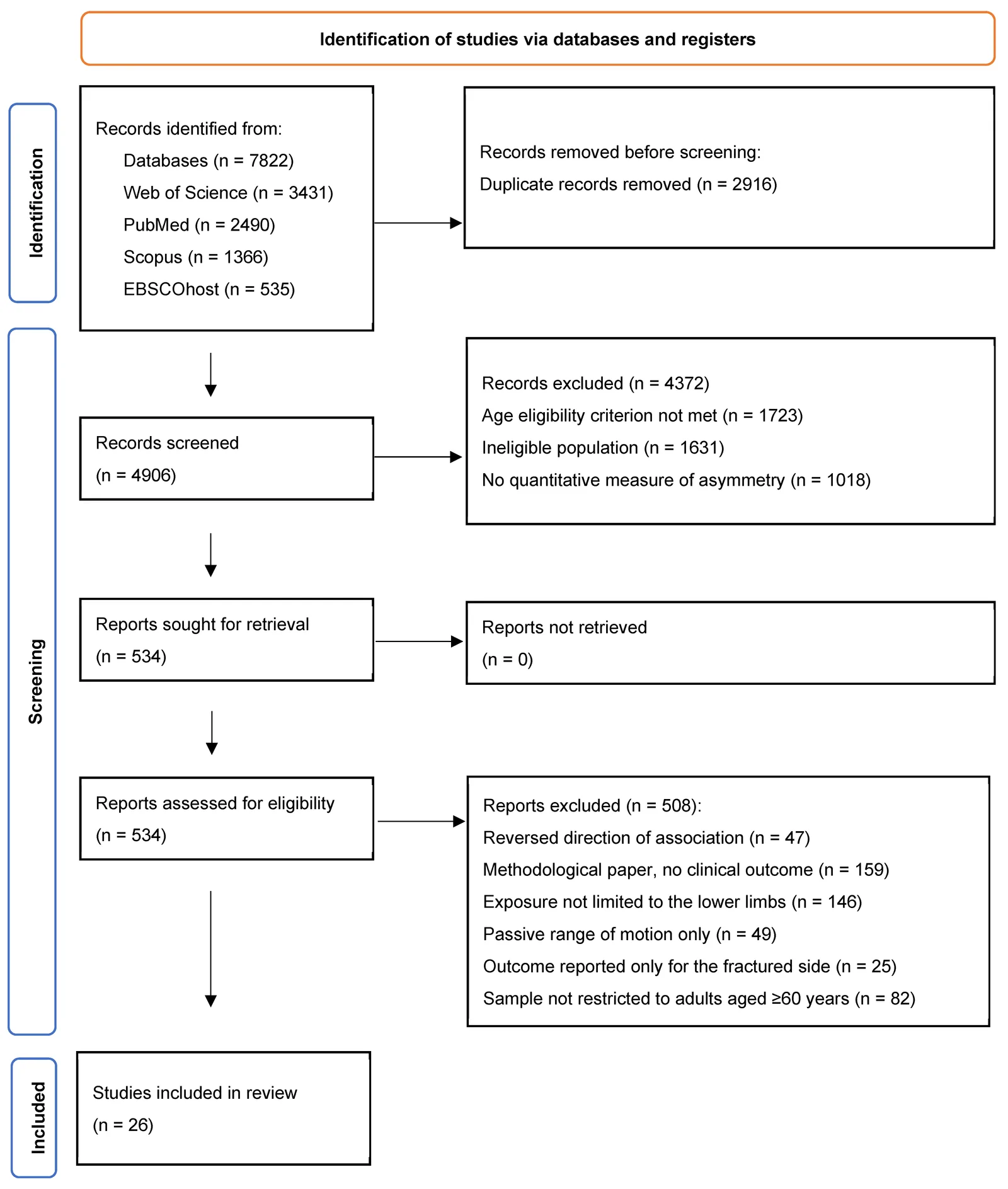
PRISMA 2020 flow diagram.

A total of 534 publications were sought for retrieval in full text, and all were obtained. After full-text assessment, 508 publications were excluded: 47 because of a reversed direction of the relationship, in which asymmetry was modeled as a consequence of age or prolonged walking; 159 because they were methodological papers that did not report any clinical outcome; 146 because the exposure concerned only coupling between the upper and lower limbs; 25 because the outcome was reported only for the side affected by hip fracture; 49 because the only measure of asymmetry reported concerned passive range of motion; and 82 in which the sample included individuals aged 19–85 years without an analysis restricted to participants aged 60 years and older. Twenty-six studies were included in the review, and all 26 were included in the synthesis. A detailed description of the stages of publication retrieval, full-text assessment and subsequent selection of eligible studies for inclusion in this systematic review is presented in Figure 1.

### 3.2. Study Characteristics

The twenty-six included studies were published between 2002 and 2026; together, they included 10,167 participants aged 60 years and older. Four studies additionally recruited younger comparison groups, which were not included in this number. The distribution of sample sizes was skewed, ranging from 24 to 5347 participants. A single prospective cohort, the Osteoporotic Fractures in Men Study [22], contributed 53% of all participants, and the two largest studies [21,22] together contributed 66%. The remaining 24 studies had a median of 95 participants.

Sixteen studies were cross-sectional, six were case–control studies and four were prospective cohorts with follow-up lasting from 10 to 24 months [18,19,20,22]. Nine studies were conducted in the United States, four in Japan, three in China, two each in South Korea and the United Kingdom, and one each in Belgium, Denmark, Finland, Portugal, Taiwan and Thailand. Twenty-three studies recruited community-dwelling individuals, one recruited residents of senior homes [29], one recruited individuals receiving long-term care [17], and another included a subgroup with clinically defined mobility limitation [15]. The characteristics of the individual studies are presented in Table 1.

**Table 1:** Characteristics of the 26 included studies, ordered by year of publication.

Study, Year (Country)	Design	*n* (Age)	Setting	Domain and Asymmetry Measure	Outcome(s)	Direction
Skelton 2002 (UK) [5]	Case–control	35 (≥65)	Community	A/B—Percentage interlimb difference in isometric and isokinetic strength and explosive power	Falls	Positive
Di Fabio 2004 (USA) [29]	Case–control	34 (77–94)	Senior homes	D/E—foot-lift asymmetry index	Fall risk; obstacle contact	Positive
Portegijs 2005 (Finland) [13]	Cross-sectional	419 (63–75)	Community	A/B—difference in leg extension power between the stronger and weaker limbs	Gait speed; tandem stance	Positive
Nakao 2006 (Japan) [30]	Cross-sectional	30 (65–89)	Community	A/C—Percentage difference in knee extension strength; thigh muscle mass asymmetry	6MWT; obstacle walking; single-leg stance	Positive
Perry 2007 (UK) [6]	Case–control	78 (≥65)	Community	A/B—Percentage difference in isometric, concentric and eccentric strength and power	Falls	No association
Carabello 2010 (USA) [15]	Cross-sectional	119 (74–78)	Community	A—knee extensor strength (1RM) and power asymmetry	SPPB; gait speed; chair rise	No association
LaRoche 2012 (USA) [31]	Cross-sectional	24 (65–80)	Community	A—knee extensor torque asymmetry	Gait asymmetry and variability	Positive
Riskowski 2012 (USA) [21]	Cross-sectional	1333 (~66)	Community	E—Symmetry Index for vertical foot force	SPPB and its components	Inverse
Straight 2016 (USA) [32]	Cross-sectional	94 (74.0 ± 5.5)	Community	A/B—Leg extension power asymmetry (%ASYM)	Composite lower-extremity performance score	Positive
James 2017a (USA) [19]	Prospective cohort	99 (≥67)	Community	E—asymmetry and variability of rhythmic ankle coordination	Incident mobility limitation (SPPB ≤ 9)	No association
James 2017b (USA) [26]	Cross-sectional	130 (67–99)	Community	E—variability and asymmetry of interlimb coordination	Mobility limitation (SPPB ≤ 9)	Positive
LaRoche 2017 (USA) [14]	Cross-sectional	36 (76.0 ± 7.6)	Community	A—knee extensor power asymmetry at 60/180/300°·s^−1^	400 m walk; stairs; chair rise	No association
Chon 2018 (South Korea) [33]	Cross-sectional	306 (65–92)	Community	A—knee extensor strength asymmetry	Posturography; BBS; TUG; SPPB	Measure-dependent
Lee 2019 (South Korea) [16]	Cross-sectional	435 (70–84)	Community (KFACS)	C—Limb Asymmetry Index of lower-limb lean mass	Gait speed; TUG; falls	Measure-dependent
Mertz 2019 (Denmark) [34]	Cross-sectional	208 (70.2 ± 3.9)	Community	A/B/C—interlimb asymmetry of mass, strength and power	400 m walk; 30 s chair stand	Measure-dependent
Gillain 2019 (Belgium) [18]	Prospective cohort	96 (65–89)	Community	D—step symmetry and its dual-task cost	Falls	Positive
McGrath 2021 (USA) [22]	Prospective cohort	5347 (73.6 ± 5.9)	Community (men)	A/B—leg extension power asymmetry	Recurrent falls; fractures	No association
Lien 2022 (Taiwan) [35]	Case–control	27 (≥65)	Community	D—gait symmetry from bilateral ankle accelerometry	Recurrent faller status	Positive
Sato 2023 (Japan) [17]	Cross-sectional	230 (≥65)	Long-term care	C—leg muscle mass asymmetry index	Gait speed	Positive
Nakanowatari 2024a (Japan) [36]	Case–control	55 (74.1 ± 6.1)	Community	E—asymmetry of contact time, peak force, loading rate and impulse	Falls in the previous year	Measure-dependent
Nakanowatari 2024b (Japan) [20]	Prospective cohort	47 (75.3 ± 5.8)	Community	E—foot loading asymmetry index	Frailty	Positive
Si 2024 (China) [37]	Cross-sectional	55 (60–75)	Community	A—isokinetic knee extension asymmetry	Gait parameters; static postural control	Positive
Castro 2025 (Portugal) [38]	Cross-sectional	60 (≥60)	Community	D/E—Continuous Relative Phase, interlimb coordination	Fall history; fear of falling	Positive
Gao 2026 (China) [39]	Cross-sectional	150	Community	D/E—51 spatiotemporal, kinematic and kinetic asymmetry features	Frailty status	Positive
Kantha 2026 (Thailand) [40]	Case–control	120 (~70)	Community	C—segmental phase angle asymmetry, muscle quality	Fall history; chair rise; TUG; FoF	Positive
Lv 2026 (China) [41]	Cross-sectional	600 (60–79)	Community	C—limb muscle mass asymmetry index	Chair rise; single-leg stance; stepping	No association

A—muscle strength and power; B—limb functional capacity; C—muscle mass, composition and quality; D—spatiotemporal gait parameters; E—kinetics, kinematics and postural control; A/B and D/E—domains merged for the synthesis; 1RM—one-repetition maximum; BBS—Berg Balance Scale; FoF—fear of falling; KFACS—Korean Frailty and Aging Cohort Study; SPPB—Short Physical Performance Battery; TUG—Timed Up and Go; 6MWT—six-minute walk test. Age is presented as reported in each source.

### 3.3. Exposure Measurement and the Lack of a Common Effect Measure

The methods used to quantify lower-limb asymmetry differed between the included studies, and the heterogeneity of the ways in which it was defined and calculated was a feature of the entire body of evidence collected. Twelve studies expressed asymmetry as the percentage difference in a bilateral measure between the stronger and weaker limbs; four used a defined index: the Symmetry Index [21], the Limb Asymmetry Index [16] or a muscle mass asymmetry index [17,41]; four used measures of interlimb coordination: rhythmic ankle coordination [19,26], Continuous Relative Phase [38] or normalized cross-correlation [35]; four used author-developed indices based on plantar loading ratios [20,36], the degree of foot lift [29] or phase angle [40]; and two used sets of gait symmetry measures: step symmetry under single- and dual-task conditions [18] and 51 spatiotemporal, kinematic and kinetic asymmetry features [39].

Considerable heterogeneity was noted in the method of exposure categorization. Three studies applied threshold values set a priori: 15% in one [37] and 20% in two [31,33]. In other studies, classification was based on tertiles [13,16] or quartiles [19,22,26], whereas in one the cut-off value was determined on the basis of ROC curve analysis [17]. The remaining seventeen studies treated asymmetry as a continuous variable. Among the studies using threshold values, there was no single common cut-off value, and no external justification was provided for the adoption of any of the thresholds applied.

Consequently, effect estimates were reported on mutually incompatible scales: as odds ratios per one-point increase in asymmetry, odds ratios or relative risks for the comparison of extreme quantiles, standardized regression coefficients, Pearson or Spearman correlation coefficients and between-group mean differences. Deriving a common effect measure was not possible, and meta-analysis was abandoned. In accordance with the SWiM guidelines [24], the direction of effect was adopted as the standardized synthesis metric, classified as positive (greater asymmetry associated with a worse outcome), no association, measure-dependent or inverse. Effect size estimates were presented alongside the adopted classification of the direction of association whenever they were available in the primary publications. In some studies, however, only *p*-values, correlation coefficients without corresponding confidence intervals or qualitative descriptions of the results were reported. This limited the ability to assess the magnitude of the observed associations and the precision of their estimates.

### 3.4. Grouping of Studies and Synthesis Method

The studies were grouped for synthesis by exposure domain according to the classification adopted a priori and then cross-tabulated by outcome.

If a study contributed data to more than one domain, its results were presented in full in the domain corresponding to the primary exposure, with a cross-reference in the secondary domain. Nakao et al. [30] and Mertz et al. [34] contributed data to domains A/B and C; LaRoche et al. [31], Chon et al. [33] and Si et al. [37] to domains A/B and D/E. The numbers of studies reported in the subsections devoted to individual domains refer to their primary classification, with the total number of assignments being 26.

The synthesis was narrative, with a structure adopted in accordance with the SWiM guidelines. Within each domain, studies were ordered by design and then by sample size, so that the most informative evidence was presented first and the weight assigned to each finding remained clear to the reader. The direction of effect was indicated in the tables across domains and outcomes (Table 2 and Table 3). These tables describe the distribution of findings and do not constitute vote counting. Conclusions were not formulated on the basis of the number of results reaching statistical significance, and statistical significance alone was not a criterion for including studies in the synthesis. Given the large differences in sample sizes, the interpretation also took into account the size of the individual studies. In domains in which the direction of effect was heterogeneous, the results from the largest studies were additionally indicated.

### 3.5. Synthesis by Exposure Domain

Table 2 summarizes the direction of effect within each of the three exposure domains used in the synthesis; the findings for the individual domains are described in Section 3.5.1, Section 3.5.2 and Section 3.5.3.

**Table 2:** Direction of effect by exposure domain.

Exposure Domain	Studies (*n*)	Positive	Measure-Dependent	No Association	Inverse
A/B: muscle strength and power	12	6	2	4	0
C: muscle mass, composition and quality	4	2	1	1	0
D/E: gait, loading, kinetics and coordination	10	7	1	1	1
Total	26	15	4	6	1

Counts describe the distribution of findings at the level of individual studies and are not a measure of the strength or certainty of evidence. In several studies, the label “positive” or “measure-dependent” rests on one or a few significant indices among several tested.

#### 3.5.1. Domain A/B: Strength and Power Symmetry

This domain included the largest number of studies and was characterized by the greatest inconsistency of results. A positive association was noted in six studies [5,13,30,31,32,37]; in two, the association was present only for selected outcome measures [33,34], whereas in four, no association was found [6,14,15,22]. The finding reported by LaRoche et al. [31], however, concerns an intermediate outcome—asymmetry and variability of support time during gait—which was taken into account. No study observed an association in the inverse direction.

The studies with the greatest statistical power did not confirm an association between lower-limb power asymmetry and the outcomes analyzed. Within the Osteoporotic Fractures in Men Study, McGrath et al. [22] followed 5347 community-dwelling men, with a mean age of 73.6 ± 5.9 years, monitoring the occurrence of falls at four-month intervals. In the fully adjusted models, membership in the highest quartile of leg extension power asymmetry was not associated with the occurrence of recurrent falls or with the risk of fracture. In the same cohort, by contrast, handgrip strength asymmetry was associated with recurrent falls (relative risk 1.20; 95% CI 1.01–1.43) and with hip fracture (hazard ratio 1.41; 95% CI 1.02–1.96), while handgrip strength asymmetry and lower-limb power asymmetry remained statistically independent (r = 0.01).

Convergent observations were presented by Perry et al. [6] in a smaller case–control study. Power asymmetry was greater in both groups of older adults than in the young control participants (*p* < 0.02–0.004). A different pattern was found for power values: fallers achieved only 79% of the power generated by non-fallers (*p* < 0.001).

A similar pattern emerged in the cross-sectional studies assessing physical performance: two studies with more extensive analyses did not show a clear association with asymmetry. LaRoche et al. [14] found no significant relationship between knee extensor power asymmetry and 400 m walk time (r = 0.16; *p* = 0.180), stair climb time (r = 0.22; *p* = 0.094) or chair rise time (r = 0.03; *p* = 0.437). At the same time, the power of both the weaker and the stronger limb showed a strong association with the level of performance (r = −0.62 for both limbs). Carabello et al. [15] also showed only a weak relationship between limb asymmetry and physical performance, whereas the associations of performance with strength and power values were more pronounced.

Positive associations were observed mainly in studies using composite indices of functional performance or more demanding tasks involving balance control. Portegijs et al. [13], in a sample of 419 healthy women aged 63–75 years, showed that high leg extension power asymmetry was associated with a 5.5-fold higher likelihood of inability to maintain the tandem stance (95% CI 2.3–13.3), which indicated a stronger association than for low power alone (2.8-fold increase; 95% CI 1.2–6.4). The association with gait speed was evident under more demanding conditions, when walking along a narrow path. Straight et al. [32] showed that leg extension power asymmetry, averaging 11.3 ± 10.5%, remained associated with the lower-extremity performance score after accounting for age and power values (standardized β = −0.18; *p* < 0.05). Correlations between asymmetry and the individual performance components ranged from r = −0.22 to −0.34. Si et al. [37], using an asymmetry threshold of 15%, found that individuals classified in the asymmetry group had slower gait (Cohen’s d = 0.97), lower cadence, shorter step and stride lengths and longer support time. Nakao et al. [30], in turn, noted a negative correlation between knee extension strength asymmetry and the distance covered in the 6MWT (r = −0.41) and a positive association with the time needed to complete the obstacle walking test (r = 0.50). Skelton et al. [5] noted greater power asymmetry in women who had experienced at least three falls compared with women without a history of falls. Moreover, the weaker limb generated 24% lower relative power per kilogram of body mass in women who fell than in women who did not fall (*p* = 0.04).

Mertz et al. [34], in a sample of 208 healthy older adults, found associations of lean mass asymmetry (r^2^ = 0.12; *p* = 0.005) and isometric strength asymmetry (r^2^ = 0.40; *p* = 0.03) with lower-limb performance, whereas dynamic strength and power asymmetry did not show such an association even though these asymmetries were clearly greater in magnitude (10–13% vs. 3 ± 2.3%). Complementary data on measurement properties were provided by Carabello et al. [15]. The intraclass correlation coefficient for asymmetry measurement was 0.87 in older adults with limited mobility and was clearly lower in healthy middle-aged adults (ICC = 0.25) and healthy older adults (ICC = 0.61). Chon et al. [33], analyzing 306 older adults classified using a 20% asymmetry threshold, found greater postural sway in the group classified as “asymmetric” during eyes-open trials, on both firm and unstable surfaces. However, no between-group differences were noted in Berg Balance Scale, Timed Up and Go or Short Physical Performance Battery scores, or in postural sway during eyes-closed trials. LaRoche et al. [31], also using a 20% threshold, showed greater asymmetry of single-limb (7.1% vs. 2.5%) and double-limb support time (7.0% vs. 4.3%) and greater variability of single-limb support time in the asymmetric group. These differences were more pronounced during walking at maximal speed.

#### 3.5.2. Domain C: Asymmetry of Muscle Mass, Composition and Quality

Despite including the fewest studies, this domain was characterized by the greatest internal consistency. It was also the only one in which the same functional outcome, “usual gait speed”, was assessed in more than one study using comparable measurement methods.

Sato et al. [17], analyzing a group of 230 individuals receiving long-term care, showed that greater lower-limb muscle mass asymmetry was associated with lower gait speed (β = −0.19; *p* = 0.002; model R^2^ = 0.261). This association persisted after accounting for age, sex, body mass index, Barthel index, handgrip strength, skeletal muscle mass index and comorbidities. ROC curve analysis indicated limited discriminative ability of the asymmetry index (AUC = 0.62; sensitivity 47.3%; specificity 81.0%; *p* = 0.014). Lee et al. [16], in a group of 435 participants in the Korean Frailty and Aging Cohort Study, noted lower gait speed in the highest tertile of the Limb Asymmetry Index compared with the lowest tertile (*p* = 0.03). No differences in Timed Up and Go scores or associations between asymmetry and the occurrence of falls were found. A convergent direction of association for lean mass asymmetry was observed by Mertz et al. [34], whose results were included in this domain as a secondary, cross-referenced finding (Section 3.4).

Kantha et al. [40] extended the scope of this domain by considering not only the quantity but also the quality of muscle tissue. In a study of 120 community-dwelling older adults, including 60 individuals with a history of falls and 60 without such a history, phase angle values did not differ between the two groups. Fallers had greater lower-limb phase angle asymmetry at a frequency of 5 kHz (*p* = 0.02). The degree of asymmetry was positively associated with faller status (r_s_ = 0.29; *p* = 0.03) and negatively associated with the chair rise test score (r_s_ = −0.32 at 5 kHz and −0.34 at 50 kHz). However, no between-group differences were found in Timed Up and Go scores or in the level of fear of falling.

Lv et al. [41], in a sample of 600 individuals aged 60–79 years, found no association between limb muscle mass asymmetry and physical performance; after correction for the false discovery rate, only the association with single-leg stance with eyes closed remained (odds ratio 1.215; 95% CI 1.017–1.452; *p* = 0.032). It should be noted that asymmetry was a secondary exploratory analysis in this study.

#### 3.5.3. Domain D/E: Asymmetry of Gait, Loading, Kinetics and Coordination

This domain contained the largest proportion of positive findings and three of the four prospective cohorts [18,19,20]. Gillain et al. [18] conducted a 24-month follow-up of 96 independently living individuals aged 65–89 years, monitoring the occurrence of falls using event diaries. During the follow-up period, 35 participants (36.5%) experienced at least one fall. In individuals who experienced a fall during the follow-up period, the baseline “cost” of step symmetry during dual-task performance was greater than in participants without subsequent falls (*p* = 0.022). Each increase of 1 percentage point in this index was associated with increased odds of a fall (OR = 1.018; 95% CI 1.002–1.033; *p* = 0.027). Nakanowatari et al. [20], in turn, followed 47 community-dwelling older adults for 10 months, 14 of whom developed frailty. In the analysis, each 1% increase in total impulse asymmetry was associated with greater odds of developing frailty (OR = 1.269; 95% CI 1.016–1.585; *p* = 0.033), whereas no analogous associations were found for the asymmetry of pressure forces.

The third prospective study showed no association between lower-limb asymmetry and the outcome analyzed, while providing important context for the interpretation of this domain. James et al. [19] followed 99 individuals aged ≥ 67 years for 12 months and showed that greater variability of rhythmic interlimb coordination of the ankles was associated with the subsequent occurrence of mobility limitation (OR = 1.88; 95% CI 1.16–3.05). No analogous association was found, however, for asymmetry in the same coordination measure (OR = 0.95; 95% CI 0.59–1.55). Mobility limitation was, in turn, associated with upper-limb coordination asymmetry (OR = 2.11; 95% CI 1.25–3.57). An accompanying cross-sectional analysis conducted in a larger sample [26] (*n* = 130) also indicated the importance of ankle coordination variability: among the participants in the quartile with the greatest variability, the odds ratios for mobility limitation were 7.38 (95% CI 2.20–24.78) and 15.40 (95% CI 4.31–55.07) relative to the quartile with the lowest variability.

Of the remaining studies, Di Fabio et al. [29] showed greater foot-lift asymmetry during obstacle avoidance and more frequent obstacle contacts in older adults at high risk of falling (*p* < 0.001), with asymmetry index values approaching zero in low-risk individuals and in young participants. Lien et al. [35] classified recurrent fallers versus non-fallers with an accuracy of 94–95% using a k-nearest neighbor model combining gait stability and symmetry from bilateral ankle accelerometry. Castro et al. [38] showed different intra- and interlimb coordination, measured with Continuous Relative Phase, in participants with a history of falls or fear of falling, particularly in hip–knee (*p* = 0.004) and hip–ankle (*p* = 0.030) couplings. Gao et al. [39], assessing 51 asymmetry indices in 150 older adults, showed differences between frailty severity grades for nine parameters after false discovery rate correction. These included spatiotemporal and kinematic measures. Nakanowatari et al. [36] found that, of the four loading asymmetry parameters analyzed, only peak force asymmetry differentiated fallers from non-fallers after accounting for confounders (*p* = 0.03; F = 4.90). This parameter had the highest measurement reliability (ICC = 0.942), whereas for the remaining three indices the ICC values did not exceed 0.75.

The largest study included in this domain showed an association in the opposite direction. Riskowski et al. [21], analyzing 1333 participants in the Framingham Foot Study, found that individuals with foot force asymmetry in the range of 3.0–9.5% achieved better Short Physical Performance Battery scores than participants with both lower and higher asymmetry. Moreover, greater asymmetry co-occurred with higher gait speed, whereas its degree decreased with age. These results are discussed in more detail in Section 3.7.

### 3.6. Synthesis by Outcome

Table 3 summarizes the direction of findings for each outcome domain; the results for falls, physical performance and mobility, and frailty are described in Section 3.6.1, Section 3.6.2 and Section 3.6.3.

**Table 3:** Direction of effect by outcome domain.

Outcome Domain	Studies (*n*)	Prospective (*n*)	Direction of Findings
Falls	10	2	6 positive, 3 no association, 1 measure-dependent
Physical performance and mobility	16	1	9 positive, 4 no association, 2 measure-dependent, 1 inverse
Frailty	2	1	2 positive

Counts describe the distribution of findings at the level of individual studies and are not a measure of the strength or certainty of evidence.

#### 3.6.1. Falls

Falls were analyzed in ten studies [5,6,16,18,22,29,35,36,38,40]. Six showed a positive association [5,18,29,35,38,40], three showed none [6,16,22], and in one, the association was limited to a single loading parameter [36]. The apparent predominance of results indicating a positive association may, however, stem from the specific study designs used. Four of the six studies reporting this direction were case–control studies [5,29,35,40], one was cross-sectional [38] and one was a prospective cohort [18]. In five of these six studies, the history of falls was ascertained retrospectively. Such a design is susceptible to reverse causation. The fall itself and its consequences may lead to changes in gait symmetry and in the pattern of limb loading; therefore, asymmetry assessed after the event cannot be interpreted as a factor known to have preceded it. Fall outcomes were also defined heterogeneously: any fall during follow-up [18], recurrent falls [22,35], at least three falls [5], falls in the previous year [36] or a clinical fall-risk classification [29]. Incident and recurrent falls differ in their determinants, and recurrent fallers typically form a more impaired group; findings obtained with these definitions are therefore not interchangeable.

Only two studies monitored the occurrence of falls prospectively, and their results were not consistent. Gillain et al. [18], following 96 participants for 24 months, showed that a greater step symmetry cost during dual-task performance was associated with the subsequent occurrence of falls. A different result was obtained by McGrath et al. [22] in a much larger sample of 5347 men, in whom falls were recorded at four-month intervals. That study found no association between leg extension power asymmetry and recurrent falls. Prospective evidence on the association of lower-limb asymmetry with the risk of falls thus remains limited to one small study indicating a positive association and one large study that did not confirm it. The available data therefore do not allow the temporal and causal relationship to be reliably established. The two prospective studies also differed in the scope of the exposure assessed, which comprised step symmetry during a dual-task condition in one study and leg extension power asymmetry in the other. Differences also concerned the outcome definition, which was the occurrence of at least one fall or of recurrent falls. Consequently, the discrepancy between the results obtained cannot be unequivocally attributed to any one of these factors.

Additional context is provided by the study by Lee et al. [16]. Despite the cross-sectional design, the authors directly analyzed the association between the Limb Asymmetry Index and fall history and did not show a relationship between these variables. In the same sample, the asymmetry index was associated with gait speed.

#### 3.6.2. Physical Performance and Mobility

Physical performance or mobility was the subject of analysis in sixteen studies. Nine showed a positive association with asymmetry; four showed no association; in two, the presence and direction of the association depended on the outcome measure used, and one found an inverse association. In cross-sectional analyses, associations with asymmetry were observed for composite performance indices, more demanding tasks involving balance control and usual gait speed. Less consistent results were obtained for single timed tests performed at a self-selected pace. Only one study of physical performance was prospective [19], and it found no association with asymmetry; the remaining evidence on performance is cross-sectional and therefore associative.

The greatest consistency of results was noted for usual gait speed. Three studies assessing muscle mass asymmetry [16,17,34], which used comparable measurement methods, i.e., bioimpedance or dual-energy X-ray absorptiometry, consistently indicated that greater asymmetry was associated with lower gait speed. Importantly, in one of these studies [17], the association persisted after adjustment for skeletal muscle mass index and handgrip strength. In contrast, the Timed Up and Go test did not detect any association in any study in which it was used [16,33,40], whereas chair rise tests yielded inconsistent results [14,15,34,40,41]. This pattern suggests that the choice of functional endpoint may influence whether an association is detected; whether this reflects a lower sensitivity of short self-paced tests, a small true association or measurement error cannot be determined from the available data (Section 4.4).

A recurring pattern in this domain was the limited additional value of asymmetry after the level of performance was accounted for. LaRoche et al. [14] showed that the power of the weaker and the stronger limb was similarly associated with the functional outcomes analyzed, whereas Mertz et al. [34] found that the parameters of the weaker limb did not predict the outcomes better than the parameters of the stronger limb. An association of asymmetry with functional outcomes independent of strength or power was shown only by Portegijs et al. [13] and Straight et al. [32]. In the study by Portegijs et al. [13], this association was evident only under the most demanding conditions of balance assessment.

#### 3.6.3. Frailty

Frailty was the outcome analyzed in two studies [20,39], both of which showed positive associations with asymmetry. Nakanowatari et al. [20], in a 10-month prospective follow-up, found that greater plantar loading asymmetry was associated with the subsequent development of frailty. Gao et al. [39], in turn, showed a cross-sectional gradient of gait asymmetry across non-frail, prefrail and frail individuals. Importantly, prefrail participants already exhibited greater asymmetry of single-limb support time and stride length than non-frail individuals. The direction of findings was consistent across both studies; however, the evidence is limited to two studies with a combined sample of 197 individuals, the prospective estimate was imprecise and based on 14 incident cases, the classification of the study by Gao et al. [39] as positive rests on nine of 51 asymmetry features tested, and the certainty of evidence was very low (Table 4).

**Table 4:** GRADE assessment of the certainty of evidence for individual exposure domain–outcome pairs.

Studies (*n*)	Risk of Bias	Inconsistency	Indirectness	Imprecision	Publication Bias	Participants (*n*)	Summary Finding	Certainty
Strength and power asymmetry (domain A/B) → falls [5,6,22]								
3	Serious ^a^	Serious ^b^	Not serious	Not serious	Not serious	5460	No consistent association; the only high-powered study found no association	⊕◯◯◯ Very low
Strength and power asymmetry (domain A/B) → physical performance [13,14,15,30,31,32,33,34,37]								
9	Serious ^c^	Serious ^d^	Not serious	Serious ^e^	Serious ^f^	1291	Divergent findings: 5 studies with positive findings, 2 with measure-dependent findings, 2 with no association; the association attenuated after adjustment for strength or power in two studies [14,34] but persisted in two [13,32]	⊕◯◯◯ Very low
Muscle mass asymmetry (domain C) → gait speed [16,17,34]								
3	Serious ^g^	Not serious	Not serious	Not serious	Not serious	873	Consistent direction in all studies	⊕◯◯◯ Very low
Muscle mass asymmetry (domain C) → other performance measures [30,40,41]								
3	Serious ^h^	Serious ^i^	Not serious	Serious ^j^	Not serious	750	Divergent findings	⊕◯◯◯ Very low
Muscle mass and quality asymmetry (domain C) → falls [16,40]								
2	Serious ^k^	Serious ^l^	Not serious	Serious ^m^	Not serious	555	One study with positive findings, one with no association; one cross-sectional and one case–control	⊕◯◯◯ Very low
Gait and loading asymmetry (domain D/E) → falls [18,29,35,36,38]								
5	Serious ^n^	Not serious	Serious ^o^	Serious ^p^	Serious ^q^	272	Predominantly positive direction (4 positive, 1 measure-dependent), but 4 of 5 studies were retrospective	⊕◯◯◯ Very low
Gait and coordination asymmetry (domain D/E) → physical performance [19,21,26]								
3	Serious ^r^	Very serious ^s^	Not serious	Not serious	Not serious	1562	Contradictory findings: one positive association, one null association, one inverse association	⊕◯◯◯ Very low
Gait and loading asymmetry (domain D/E) → frailty [20,39]								
2	Serious ^t^	Not serious	Not serious	Serious ^u^	Not serious	197	Consistent direction; one prospective study and one study showing a gradient across three frailty grades	⊕◯◯◯ Very low

The assessment started at a low level of certainty (⊕⊕◯◯) appropriate for observational evidence [25]. “Serious” denotes downgrading by one level and “very serious” by two levels. Explanations: ^a^ Two case–control studies used retrospective fall reports, unvalidated case definitions and purposive samples [5,6]. ^b^ Positive finding in one small study [5], but null findings in another [6] and a large prospective cohort (*n* = 5347) [22]. ^c^ All nine studies were cross-sectional; seven had high or moderate risk of bias, and none justified sample size [14,30,31,32,33,34,37]. ^d^ Five positive, two measure-dependent and two null findings; adjustment attenuated associations in two studies [14,34], but not in two others [13,32]. ^e^ Eight studies had ≤306 participants; confidence intervals were scarce and pooling infeasible. ^f^ Multiple indices were tested without correction; positive findings clustered in small studies (*n* = 24–94) [30,31,32,37]. ^g^ All three studies were cross-sectional; only one adjusted for muscle mass and grip strength [17]. ^h^ One high-risk cross-sectional study [30] and one retrospective, purposively sampled case–control study [40]. ^i^ Two small studies were positive [30,40]; the largest (*n* = 600) was mostly null after FDR correction [41]. ^j^ Two studies had ≤120 participants; confidence intervals were largely unavailable [30,40]. ^k^ Both studies used retrospectively self-reported falls [16,40]. ^l^ Positive finding in the case–control study [40], but null in the larger cross-sectional study (*n* = 435) [16]. ^m^ Only two studies (*n* = 555), reporting *p*-values or correlations without confidence intervals. ^n^ Four studies assessed falls retrospectively; two had high risk of bias [35,38]. ^o^ Surrogate outcomes included fall-risk classification, obstacle contacts [29] and a combined gait model unable to isolate asymmetry [35]. ^p^ Only 272 participants across studies (*n* = 27–96); no pooled estimate. ^q^ Multiple testing yielded isolated significant findings in small studies [36,38]. ^r^ Two studies were cross-sectional [21,26]; the prospective study had incomplete follow-up [19]. ^s^ Very serious inconsistency: positive [26], null [19] and inverse [21] findings; the largest study (*n* = 1333) showed an inverse association. ^t^ One small prospective study lacked adjustment for loading parameters [20]; the other was cross-sectional [39]. ^u^ Only 197 participants and 14 incident frailty cases; OR 1.269 (95% CI 1.016–1.585) per 1% impulse asymmetry [20].

### 3.7. Threshold and Non-Monotonic Patterns (Post Hoc Exploratory Analysis)

This analysis was not prespecified in the registered protocol and was undertaken after the data had been examined; its results are hypothesis-generating only. Three observations from studies of different designs and covering different exposure domains suggest the possibility that the association between lower-limb asymmetry and physical performance is neither linear nor unequivocally unfavorable. Such a pattern is relevant to the interpretation of the previously presented results indicating no association.

The most direct evidence was provided by Riskowski et al. [21], who, in the largest cross-sectional sample included in the review, observed a curvilinear relationship: a moderate level of foot force asymmetry co-occurred with better physical performance. LaRoche et al. [14] did not show an independent association of asymmetry itself with functional outcomes. Participants characterized by both low power and high asymmetry walked 400 m 26% more slowly than individuals with high power and low asymmetry (*p* = 0.015), and their stair climb time was 31% longer (*p* = 0.033). This result suggests the potential importance of the co-occurrence of asymmetry and low power. Portegijs et al. [13] observed an association with asymmetry during walking along a narrow path and during tandem stance, i.e., in tasks placing greater demands on postural control.

Taken together, the results are consistent with a hypothetical threshold model, according to which interlimb asymmetry may become functionally relevant only after a certain level has been exceeded, particularly in individuals with reduced neuromuscular capacity or during tasks that increase the demands on balance control. Such a model could explain the lack of association observed in samples of healthy, high-functioning older adults in which asymmetry was analyzed as a continuous main effect. Because none of the included studies was designed to test such a mechanism directly, this model should be treated solely as a hypothesis based on the pattern of results emerging from the synthesis.

The thresholds used in the literature provide no empirical guidance as to where such a boundary lies. The cut-off values of 15% and 20% adopted by Si et al. [37], LaRoche et al. [31] and Chon et al. [33] were adapted from sports medicine practice [10,11] rather than derived from data on older populations, whereas the only data-derived cut-off value within the review [17] achieved an area under the curve of 0.62, which has limited discriminative value.

### 3.8. Risk of Bias in Included Studies

**Figure 2:**
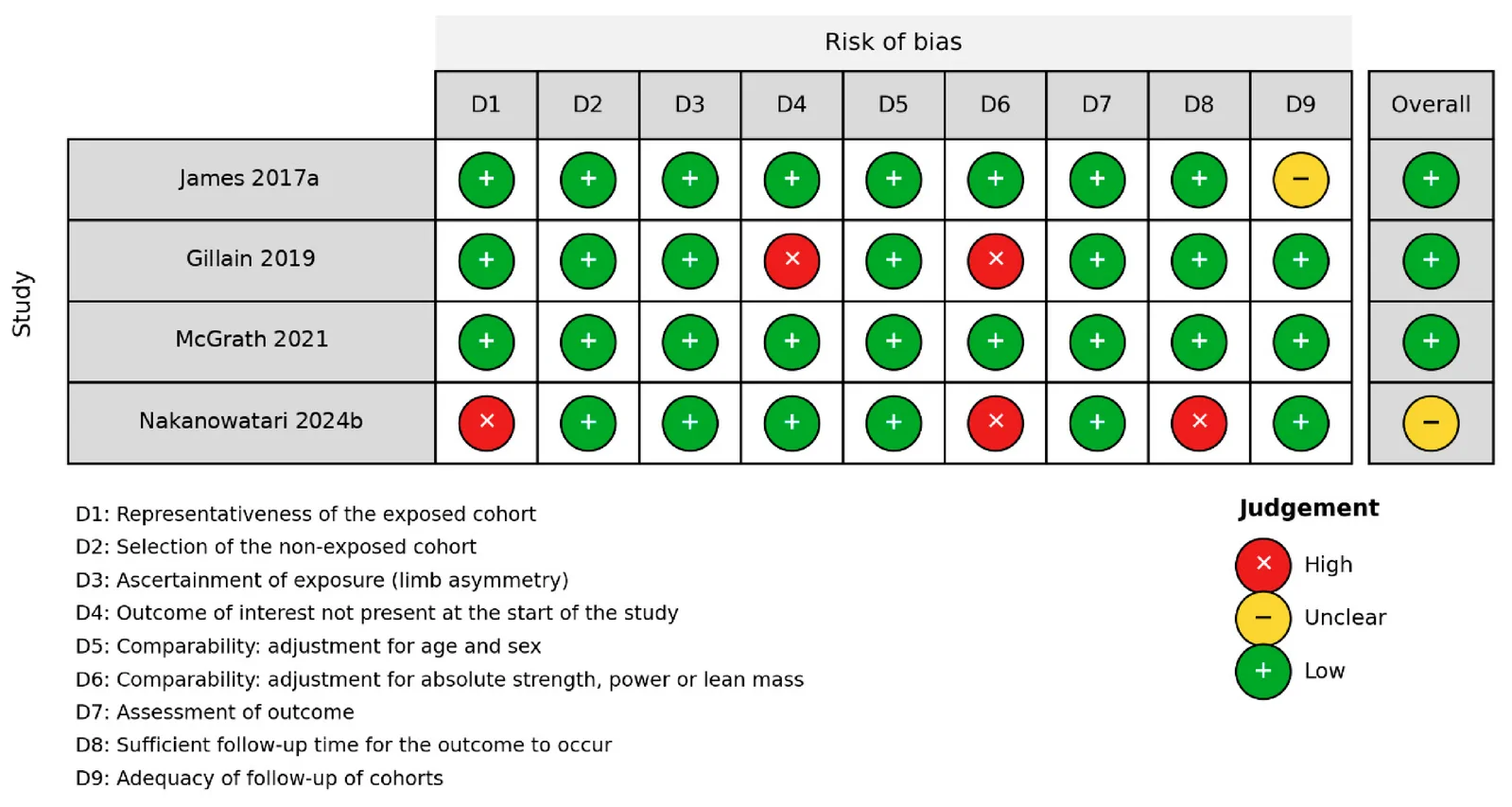
Risk of bias in prospective cohort studies [18,19,20,22].

**Figure 3:**
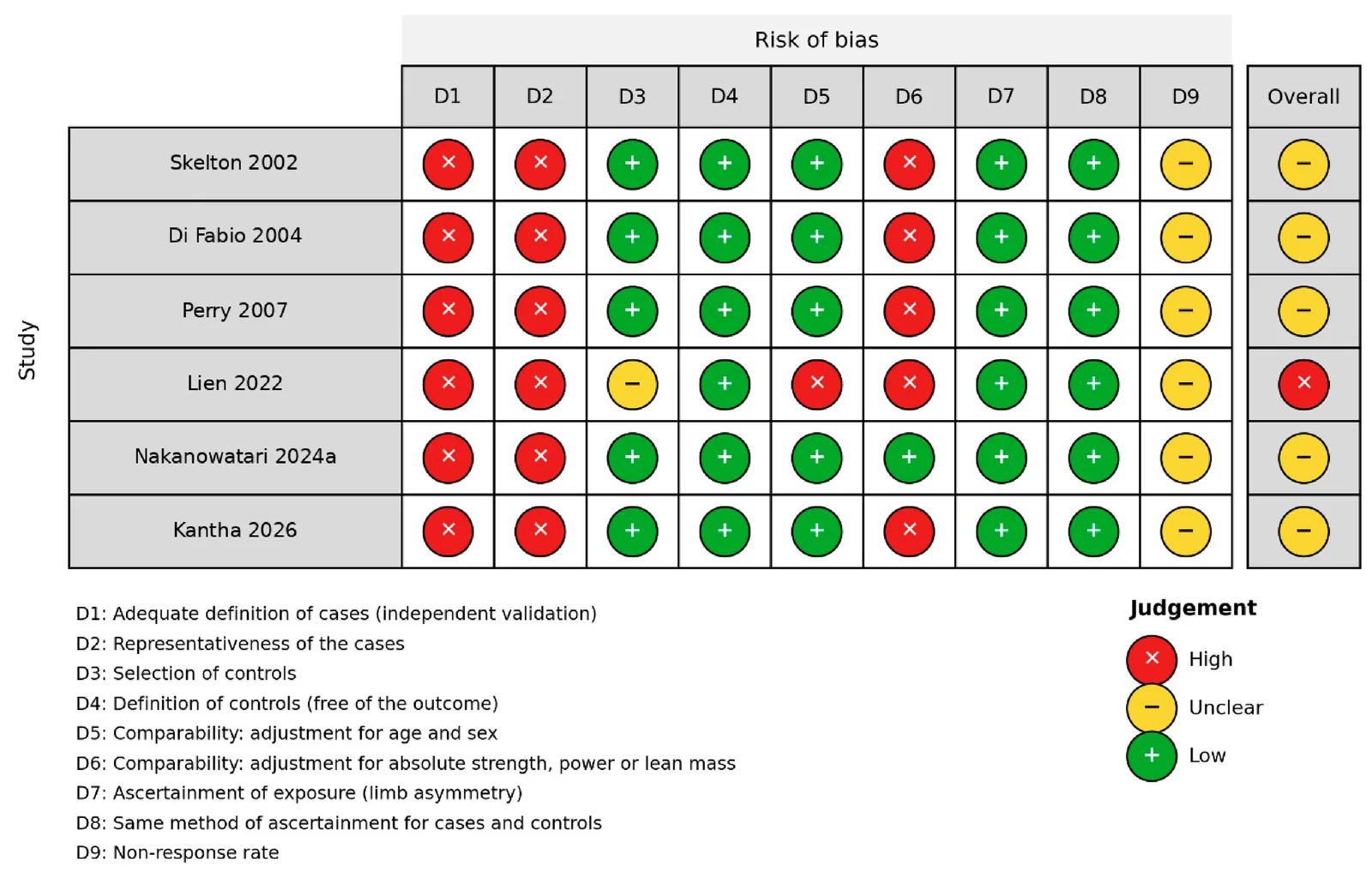
Risk of bias in case–control studies [5,6,29,35,36,40].

**Figure 4:**
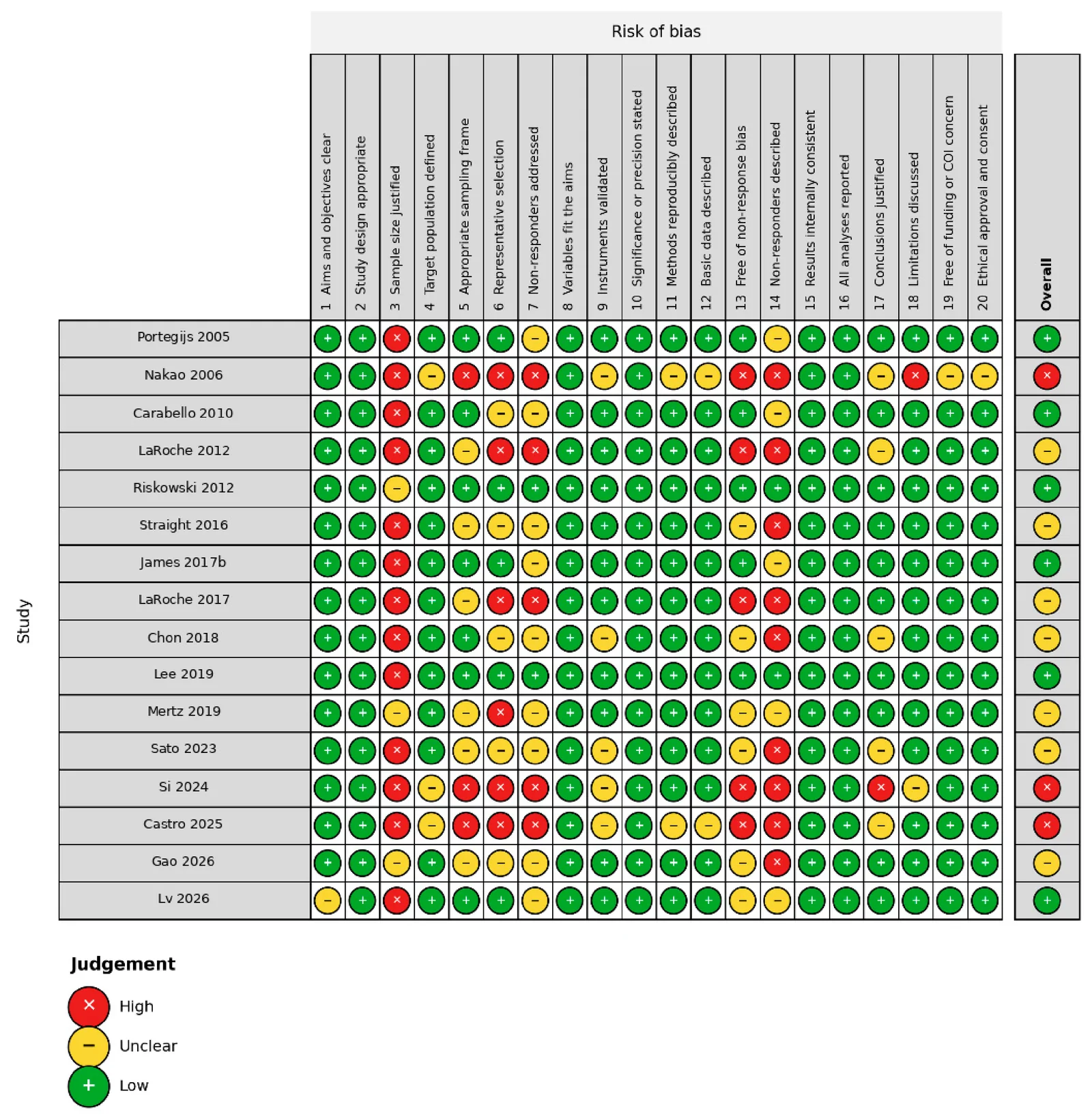
Risk of bias in cross-sectional studies [13,14,15,16,17,21,26,30,31,32,33,34,37,38,39,41].

**Figure 5:**
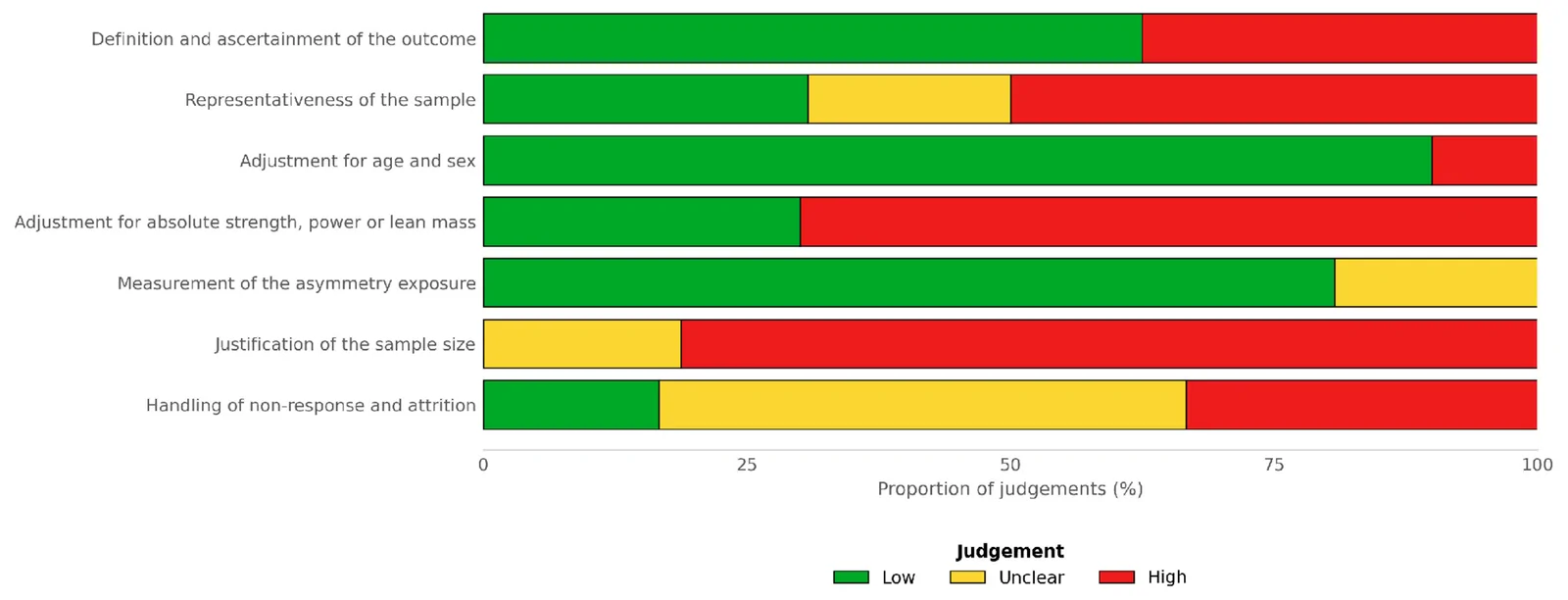
Distribution of risk of bias judgments.

The item-level ratings obtained using the tools for the 26 studies are presented in Figure 2, Figure 3 and Figure 4, and the distribution of ratings by substantive domain is shown in Figure 5. No study was excluded from the synthesis on the basis of the risk of bias assessment.

#### 3.8.1. Prospective Cohorts and Case–Control Studies

Of the four prospective cohorts, three received ratings corresponding to a low risk of bias and one to a moderate risk (Figure 2). The study by McGrath et al. [22] was the only study in the review to meet all nine assessment criteria. James et al. [19] did not meet one criterion, concerning the completeness of follow-up. In the study by Gillain et al. [18], the absence of the outcome of interest at the start of follow-up was not demonstrated, because previous falls were not an exclusion criterion; moreover, locomotor capacity was not accounted for in the analyses. Nakanowatari et al. [20] received a rating corresponding to a moderate risk of bias, which resulted from a small, purposively selected sample, a 10-month follow-up period that was relatively short for assessing incident frailty, and the lack of adjustment for loading parameters.

None of the six case–control studies reached the threshold corresponding to a low risk of bias (Figure 3). Three assessment items remained problematic in all studies in this group. The case definition was not independently validated: faller status was ascertained retrospectively on the basis of self-report, without confirmation from medical records. No study demonstrated the representativeness of the cases. The samples comprised 27 to 120 participants and were based on purposive recruitment. Of the six studies, only one [36] adjusted for the level of neuromuscular capacity.

A high risk of bias was found in one study [35], which did not describe how the control group was selected. The classification accuracy of 94–95% reported in that study, obtained with a k-nearest neighbor algorithm, was estimated in a sample of 27 individuals.

#### 3.8.2. Cross-Sectional Studies

Of the sixteen cross-sectional studies, six received a rating corresponding to a low risk of bias [13,15,16,21,26,41], seven to a moderate risk [14,17,31,32,33,34,39] and three to a high risk [30,37,38] (Figure 4). The highest methodological quality was found in studies using data from existing population-based cohorts: Riskowski et al. [21] (19.5/20), Lee et al. [16] (19/20), and Portegijs et al. [13] and James et al. [26] (18/20 each).

The ratings of the individual tool items showed considerable heterogeneity. Six criteria relating to the transparency of reporting, namely a clear statement of the aims, appropriateness of the study design, appropriate selection of variables, the method of determining statistical significance, internal consistency of the results presented and completeness of reporting of the planned analyses, were met in almost all studies. As a result, these criteria had little ability to distinguish between studies in terms of methodological quality in the analyzed set.

Potential sources of bias were concentrated in several areas. No study provided a full sample size justification: thirteen provided none, and three provided only a partial justification. The handling of non-response was described satisfactorily in only two studies [16,21], whereas nine provided no information on individuals who did not take part in the study. The representativeness of the sampling process was judged sufficient in five studies and insufficient in six. In the latter, the participants were volunteers recruited during health-promotion classes or patients from a single center.

The criterion relating to the prior validation of the measurement methods used requires separate discussion. The equipment used for the quantitative assessment of asymmetry was generally adequately validated. In five studies, the rating was lowered to partially met because an additional transformation, whose validity had not previously been confirmed, was applied to the validated measurement. This concerned the use of asymmetry thresholds of 15% or 20% taken from the sports medicine literature [31,33,37], as well as a cut-off value determined internally on the basis of ROC curve analysis with limited discriminative ability (AUC = 0.62) [17]. In addition, in one study [37], the conclusions were judged to go beyond the scope of the results obtained, because a causal interpretation was proposed on the basis of cross-sectional data.

#### 3.8.3. Cross-Cutting Patterns and Their Implications for the Interpretation of Results

The combined summary of the ratings obtained with both tools indicates that the potential sources of systematic bias were not evenly distributed across the analyzed body of evidence (Figure 5). The measurement of exposure itself was rated most favorably: 81% of the ratings indicated a low risk of bias, which was related to the frequent use of objective measurement methods. The main methodological limitations of this literature concern study design and the analytical context in which the measurements obtained were interpreted. Adjustment for the level of muscle strength, power or mass was applied in only three of the ten studies assessed in this respect. Moreover, none of the cross-sectional studies provided a full justification of the sample size, the representativeness of the sample was judged satisfactory in only 31% of ratings, and appropriate handling of non-response or loss to follow-up was found in 17% of ratings.

The lack of adjustment for the level of neuromuscular capacity means that, in most studies, the association attributed to asymmetry cannot be clearly separated from the potential influence of the reduced capacity of the weaker limb. This observation is consistent with the results presented in Section 3.6.2: in studies applying such an adjustment, the association between asymmetry and the outcome analyzed weakened or disappeared [14,34], whereas it persisted in only two studies [13,32], and in one of them it was evident in the tasks placing the greatest demands on postural control. In all six case–control studies, information on falls was obtained retrospectively on the basis of self-report. Such an assessment does not allow the temporal sequence to be reliably determined. The reliability of asymmetry measurement was reported in only two studies [15,36], and the results obtained indicated that it depended on the measure used and the population studied. In one study, the intraclass correlation coefficient ranged from 0.25 to 0.87 [15], whereas in the other it reached 0.942 for one of the parameters, while for the remaining three it did not exceed 0.75 [36].

The methodological limitations identified may affect the estimates in opposite directions. Retrospective ascertainment of falls and the lack of blinding of asymmetry assessment to faller status may favor the detection of an association and lead to an overestimation of its observed magnitude. Conversely, limited reliability of exposure measurement and its dichotomization using threshold values transferred from other populations may lead to non-differential misclassification, potentially attenuating the observed associations and shifting the estimates toward the null. On the basis of the available data, the direction and magnitude of these biases cannot be reliably determined; therefore, both results indicating the presence of an association and null results require cautious interpretation.

At the same time, a pattern was observed indicating that the evidence for the hypothesized association was weaker in studies characterized by greater methodological rigor. The three studies that received the highest ratings in the methodological quality assessment—McGrath et al. [22] (9/9), Riskowski et al. [21] (19.5/20) and Lee et al. [16] (19/20)—did not confirm the hypothesized direction of the association or showed a curvilinear association. In contrast, four of the six positive findings concerning falls came from case–control studies, none of which met the criteria for a low risk of bias. The distribution of results does not allow the direction or magnitude of the effects of systematic bias to be determined quantitatively, but it indicates that positive associations were more often observed in studies susceptible to biases that could lead to their overestimation. This was the basis for downgrading the certainty of evidence in the risk of bias domain for all eight exposure–outcome pairs presented in Table 4.

### 3.9. Reporting Biases

An assessment of publication bias using a funnel plot or appropriate statistical tests was not possible because of the lack of a common and comparable effect measure. Nevertheless, two areas requiring attention can be identified in the collected evidence. The distribution of the direction of results was uneven: 15 of 26 studies indicated a positive association, whereas six found no association. This pattern should be interpreted with caution, especially as the largest and more methodologically rigorous studies did not confirm the expected direction of the association [21,22]. Some studies analyzed multiple asymmetry parameters simultaneously—as many as 51 in one study [39] and four to six in several others—with only some of the associations assessed reaching statistical significance. In studies applying correction for multiple testing [39,41], the number of results retaining significance decreased. Consequently, in studies without such a correction, selective reporting of significant results from among a larger number of analyses performed cannot be ruled out, which indicates a potential risk of bias due to selective reporting in this literature. In several studies, the classification as “positive” or “measure-dependent” rested on one or a few significant indices among several tested: for example, one of four loading parameters [36], total impulse but not pressure-force asymmetry [20], and nine of 51 features [39]. Such isolated positive findings, derived from small studies without correction for multiple testing, should be regarded as hypothesis-generating.

### 3.10. Certainty of Evidence

The certainty of evidence was assessed using the GRADE approach [25] separately for each exposure domain–outcome pair. In accordance with the principles of the method, all pairs started at a low level of certainty, as appropriate for bodies of observational evidence and were then downgraded in the domains of risk of bias, inconsistency, indirectness, imprecision and publication bias. No pair met the criteria for upgrading, because none showed a large effect size, a dose–response gradient or a situation in which the confounders accounted for would act against the observed effect. The results of the assessment are presented in Table 4.

The certainty of evidence was rated as very low for all eight exposure domain–outcome pairs. The pairs differed, however, in the number of domains in which the rating was downgraded. The most favorable profile was found for the association of lower-limb muscle mass asymmetry with usual gait speed [16,17,34], which was downgraded only in the risk of bias domain: three studies showed a consistent direction of effect, one of them [17] adjusted for skeletal muscle mass index and handgrip strength, the measurement equipment used was comparable, and the total number of participants (*n* = 873) did not justify downgrading in the imprecision domain. The remaining seven pairs were downgraded in at least two domains. The difference between the bodies of evidence is thus a matter of degree and concerns the number and weight of the limitations, not the final level of certainty, which in no case reached the moderate level. For this pair, the exclusively cross-sectional nature of the entire body of evidence precludes any inference about the temporal sequence. This limitation was accounted for through the low starting level for observational evidence and in the risk of bias rating. The evidence for this pair concerns the association with concurrent gait speed and cannot show whether muscle mass asymmetry predicts future decline in physical performance.

Inconsistency of results was the main limitation for the pairs involving strength and power asymmetry in relation to falls [5,6,22] and gait and coordination asymmetry in relation to physical performance [19,21,26]; in the latter pair, it was rated as very serious. In both cases, the largest study in the given set showed an association in a direction different from that observed in the smaller studies. Such a pattern undermines the assumption of a consistent and homogeneous effect and indicates the need for particular caution when translating the results of this review into practical recommendations. Overall, the very low certainty of evidence originates mainly from limitations of the primary studies, whereas review-level constraints contributed mainly to the judgements in the imprecision and publication bias domains.

## 4. Discussion

This review indicates that the association of lower-limb asymmetry with falls and performance in older adults cannot be described by a single common effect. The importance of asymmetry depended on the construct measured and the functional task. For strength and power asymmetry, the results were heterogeneous, and the largest prospective cohort did not confirm an association of power asymmetry with recurrent falls [22]. A more consistent direction was observed for muscle mass asymmetry and gait speed [16,17,34]. In turn, selected measures of gait and loading asymmetry showed associations with subsequent falls or the development of frailty, but they came from small cohorts [18,20]. All of these findings rest on very low-certainty evidence. The body of evidence as a whole therefore does not justify treating asymmetry as a universal, independent prognostic factor or as a standalone target for fall prevention.

The large cohort study on power asymmetry provides the most informative data on the association of the exposure with recurrent falls, but its results do not permit inferences about the importance of muscle mass or coordination asymmetry [22]. Similarly, greater consistency of results in a small group of studies does not automatically mean greater certainty of evidence. The GRADE assessment presented in this review indicates very low certainty, which limits both inferences about the independence of the observed associations and their clinical application [25].

### 4.1. Strength and Power Asymmetry and Muscle Capacity

The results of the strength and power domain challenge the assumption that a greater difference between the limbs must mean poorer performance. McGrath et al. [22] showed no association of leg extension power asymmetry with recurrent falls in a cohort of 5347 men. In the study by Perry et al. [6], fallers were characterized by lower power, whereas interlimb asymmetry in power generation did not differentiate older adults according to their history of falls. Similarly, LaRoche et al. [14] and Carabello et al. [15] showed stronger associations of performance with the level of strength or power than with their interlimb imbalance. These results support considering the magnitude of the available muscle capacity separately from the way it is distributed between the lower limbs.

Bean et al. [42], studying 45 older adults with mobility limitation, found that lower-limb power explained 2–8 percentage points more of the variance in functional outcomes than strength in adjusted models. Although the study did not directly address asymmetry, its results indicate that the interpretation of interlimb differences should take into account the level of muscle power. Combined with the observations of LaRoche et al. [14] and Carabello et al. [15], this suggests that some of the differences in functional performance attributed to asymmetry may in fact reflect a limited ability to generate muscle power rapidly. These results do not, however, rule out the possibility that asymmetry provides additional information in tasks imposing greater functional demands.

The same relative asymmetry index may describe very different limb profiles. For example, knee extension torques of 100 and 80 N·m and of 50 and 40 N·m both yield 20% asymmetry when the stronger limb is used as the reference, although the second profile reflects bilateral weakness. Similar values on both sides therefore do not rule out a functional deficit, and greater asymmetry may result from a deficit of the weaker limb as well as from preserved performance of the stronger limb. The relative asymmetry index alone cannot distinguish between these scenarios, which carry different clinical meanings. For this reason, associations between asymmetry and the outcome analyzed should be interpreted taking into account the absolute values obtained for both limbs and the method used to normalize the measurement [11,14,15,34].

This does not mean that strength or power asymmetry has no significance. Portegijs et al. [13] showed associations in tasks requiring increased balance control, and Straight et al. [32] noted an association with a composite performance score after accounting for age and power. The magnitude of the latter estimate, however, indicates a limited association. Both observations suggest that its additional value may depend on the task and on the participants’ level of capacity. They do not, however, constitute evidence that a targeted reduction in asymmetry improves function independently of an increase in strength or power.

Including quantitative muscle parameters in the analysis is of particular importance when assessing whether asymmetry provides additional information beyond the level of muscle capacity; however, it requires a precise definition of the research question being analyzed. The asymmetry index is derived from the same measurements that describe the performance of both limbs; simultaneously entering related variables may hinder the interpretation of the model. Persistence of an association after adjustment does not prove causality, and its attenuation does not in itself identify a mechanism. The available data allow us to assess whether the measurement of asymmetry provides information beyond that resulting from the level of muscle strength or power. However, the results to date do not allow this question to be resolved unequivocally [13,14,32,34].

### 4.2. Muscle Mass and Quality Asymmetry and Physical Performance

The most consistent direction of association in the structural domain concerned greater muscle mass asymmetry and lower gait speed [16,17,34], although all three studies were cross-sectional and the certainty of this evidence was very low. The observations came from different settings, including individuals living independently and those requiring long-term care. In the study by Sato et al. [17], the association persisted after accounting for demographic and functional characteristics, including skeletal muscle mass index and handgrip strength. This may suggest that the way muscle mass is distributed between the limbs provides information on functional performance that is not fully reflected by its total amount. The available data do not, however, allow the direction of this relationship to be established. It is not known whether an uneven distribution of muscle mass precedes a deterioration in gait speed or is partly a consequence of earlier limitations in physical activity and uneven limb loading.

The distinction between muscle mass and the capacity to generate force is supported by the Health ABC study. Goodpaster et al. [43], during a three-year follow-up of 1880 older adults, showed that lower-limb strength declined approximately three times faster than lean mass; maintaining or increasing mass did not prevent the decline in strength. This result provides context for the discrepancy between the structural and functional domains in this review. Mass asymmetry should not be expected to be equivalent to strength or power asymmetry, because aging also involves changes in the properties and function of muscle.

The statistical association did not translate into a strong ability to identify functional limitations. In the study by Sato et al. [17], the area under the ROC curve was 0.62, with a sensitivity of 47.3% and a specificity of 81.0%. These results limit the usefulness of the index studied as a standalone screening test. Moreover, Lee et al. [16] did not show analogous associations for Timed Up and Go or fall history, and in the study by Lv et al. [41], only a selected association with single-leg balance remained after correction for multiple testing.

The significance of the association with gait speed should nevertheless be appreciated in light of its documented prognostic value. In a pooled analysis of seven studies including 27,220 individuals aged 65 years and older, Perera et al. [44] showed that higher baseline gait speed was associated with a lower incidence of new mobility limitations and dependence in basic activities within three years. This provides a rationale for choosing gait speed as a clinically relevant outcome in analyses of asymmetry.

### 4.3. Gait and Loading Asymmetry in Relation to Falls and Frailty

Evidence suggesting possible prognostic significance of gait asymmetry is provided by the study by Gillain et al. [18], in which a greater step symmetry cost during dual-task performance preceded the occurrence of falls. The reported odds ratio of 1.018 per increase of one percentage point in the index (95% CI 1.002–1.033) should be interpreted taking into account the unit and the actual range of exposure values. It is possible that dual-task conditions reveal deficits in locomotor control that remain undetected during free walking.

Most of the remaining data on falls concerned the history of events or comparisons of groups distinguished according to faller status [5,6,16,35,36,38,40]. In such studies, changes in the gait pattern may have preceded a fall, but they may also have been a consequence of injury or activity limitation. Moreover, obstacle contact and membership in the high-risk group, assessed by Di Fabio et al. [29], are not equivalent to a prospectively recorded fall. A joint interpretation of these results may be useful for characterizing the phenomenon studied; nevertheless, it does not justify attributing equivalent prognostic value to individual findings.

Asymmetry should also be distinguished from variability and coordination of movement. James et al. [19] showed an association of ankle coordination variability with subsequent mobility limitation but did not confirm it for the asymmetry of this coordination (OR = 0.95; 95% CI 0.59–1.55). The associations concerning variability in the accompanying cross-sectional analysis [26] therefore cannot serve as a direct confirmation of the asymmetry hypothesis. Similarly, the high accuracy of the model combining gait stability and symmetry in the small sample of Lien et al. [35] does not allow its performance to be attributed to asymmetry alone or regarded as confirmed evidence of its ability to predict future events. Independent validation and a comparison of models with and without this index are needed.

The importance of this distinction is supported by the broader prospective literature. Verghese et al. [45], analyzing 597 individuals aged ≥70 years, showed that selected gait parameters, including stride length variability and swing time variability, were associated with the subsequent occurrence of falls, even after accounting for clinical characteristics and the results of standard gait and balance tests. These observations are consistent with the findings of James et al. [19] indicating the potential importance of variability, although they relate to a different clinical outcome. Separate modeling of both parameters is necessary to determine which of these dimensions of locomotor control accounts for the observed prognostic signal.

The evidence concerning the association of asymmetry with frailty is preliminary. Nakanowatari et al. [20] showed that total impulse asymmetry was associated with the subsequent development of frailty. The estimated odds ratio (OR = 1.269 per unit of exposure as defined by the authors; 95% CI 1.016–1.585) has limited precision and requires confirmation in larger cohorts. The cross-sectional differences between frailty categories observed by Gao et al. [39], in turn, provide evidence consistent with the existence of such an association. Moreover, the observed gradient between performance categories is not equivalent to demonstrating that an increase in asymmetry in an individual leads to the progression of frailty.

### 4.4. Task Specificity and the Post Hoc Non-Linear Association Hypothesis

The heterogeneity of results may partly stem from the dependence of the importance of asymmetry on the demands of the task performed. The greater differences in gait parameters observed at maximal speed [31], the associations found during walking along a narrow path and in the tandem stance [13], and the results obtained under dual-task conditions [18] are consistent with the hypothesis that associations with asymmetry may become more apparent as task difficulty increases. At the same time, the lack of association in the Timed Up and Go test [16,33,40] should not be interpreted as evidence of its insufficient sensitivity. Such a result may also stem from the small magnitude of the effect studied or from measurement error. The available data do not currently allow us to determine which of these explanations best accounts for the observed discrepancies.

Outcome measures also differ in the demands they place on the two limbs. Usual gait speed reflects repeated alternating loading over a longer distance; the Timed Up and Go test combines a sit-to-stand transfer, turning and walking at a self-selected pace, so its total time may be influenced by partly bilateral components; and the SPPB is a composite score with ceiling effects in high-functioning samples. Tasks with greater balance demands or with an additional cognitive load may increase reliance on the weaker limb or on interlimb coordination. These differences may partly explain why associations were detected for some outcomes. Future studies should therefore select outcome measures according to the hypothesized mechanism. Additional context is provided by the 2025 study by Kim et al. [46], conducted in a group of 303 older adults. The authors showed that step width variability differentiated the study groups more clearly during walking at a speed 20% higher than the preferred speed. This observation is consistent with the hypothesis that increasing the demands of the task may reveal impairments of locomotor control that are less apparent during free walking. It should be emphasized, however, that the groups were distinguished on the basis of SPPB scores. The results obtained therefore relate to the ability to distinguish between levels of performance used as a risk indicator. Together with the observations of LaRoche et al. [31] and Gillain et al. [18], this provides a rationale for further comparison of different testing conditions, but it does not justify assuming that tasks of greater difficulty automatically have greater clinical value.

The study by Riskowski et al. [21] further challenges the assumption that all asymmetry has a linearly unfavorable association with performance: moderate foot force asymmetry co-occurred with a better SPPB score than lower or higher values. The observed pattern may result from movement organization or from confounding factors. Its interpretation should remain limited to the parameter and population studied.

A similar possibility of a non-monotonic relationship has emerged in the literature on gait variability. Brach et al. [47] found that among older adults walking at a speed of at least 1.0 m/s, both low and high step width variability were more often associated with a history of falls than intermediate values. This is a conceptual analogy to the results of Riskowski et al. [21]. Both observations caution against the assumption that minimizing every deviation of the gait pattern is beneficial.

The hypothesis of an interaction between asymmetry and reduced muscle capacity also requires caution. The poorer results of individuals with both low power and high asymmetry in the study by LaRoche et al. [14] do not allow the effect of power to be separated from the effect of asymmetry or an interaction to be confirmed without an appropriate statistical analysis. The model in which asymmetry becomes relevant after a certain level has been exceeded is a post hoc hypothesis generated by the synthesis, not a finding; it was not prespecified, has not been tested in any included study and should not be used to guide clinical decisions. Current data do not define such a threshold. The cut-off values of 15% and 20% used in some studies [31,33,37] were not sufficiently justified in this review as universal clinical thresholds for older adults.

### 4.5. Limitations of the Evidence and Certainty of Interpretation

The fundamental limitation of the available body of evidence is the predominance of cross-sectional and case–control studies. Limitations also concern the prospective studies, in which the extent of control for potential confounders was heterogeneous and the small number of events in the smaller cohorts limited the possibility of reliable multivariable modeling [18,19,20].

Potential effect modifiers could not be examined formally because each subgroup contained too few studies. Sex was unevenly represented: the largest cohort, which found no association, enrolled only men [22], whereas several positive findings for strength and power asymmetry came from samples of women [5,13,30,31,32]. Sex and measurement domain are therefore confounded in this body of evidence, and whether the associations differ between women and men remains unknown. Subgroup-specific estimates by sex and care setting therefore remain an important evidence gap. These limitations are not specific to gerontology. In a review of 53 papers, Parkinson et al. [48] identified 12 methods of calculating strength asymmetry, of which only four were not affected by limitations related to the choice of the reference limb. The commonly used thresholds of 10–15% did not always have adequate empirical justification. This summary reinforces the reservations about transferring thresholds from other populations to the assessment of older adults in studies [31,33,37]. The agreement between the reviews concerns this methodological issue.

The method of calculating asymmetry affects its value and classification. For example, for values of 100 and 80 units, asymmetry equals 20%, 25% or 22.2%, depending on whether the reference is the stronger limb, the weaker limb or the mean of both limbs. Indices that take the dominant side into account may additionally differ in sign [11,48]. This means that identical percentage thresholds may lead to different classifications of participants, and values adopted in sports medicine cannot be directly transferred between indices and populations. In this review, 12 studies used the percentage difference between the stronger and weaker limbs, whereas the remaining studies applied indices of different construction, including coordination measures and author-developed calculation methods. As a result, the same nominal asymmetry thresholds were not equivalent across studies.

On the basis of the available data, the direction of the effects of individual sources of systematic bias cannot be determined. Measurement error and categorization of the exposure may reduce the ability to detect a true association, although the direction and scale of this influence depend on the nature of the error and the analytical model applied. Consequently, a lack of statistical significance should not be equated with demonstrating the absence of an effect or equivalence. At the same time, isolated positive results obtained within analyses covering multiple indices require cautious interpretation, especially when no correction for multiple testing was applied. The reduction in the number of results retaining significance after such a correction was applied in the studies by Gao et al. [39] and Lv et al. [41] indicates the practical importance of this problem.

Residual confounding remains an important limitation. Neuromuscular parameters were accounted for in only three of the ten studies assessed, whereas other potential confounders, including unilateral joint pain, osteoarthritis, previous injuries, comorbidities and physical activity, were inconsistently controlled for. In studies of gait and loading asymmetry, footwear may have been an additional source of variability, as it affects gait biomechanics and may be associated with fall risk [49,50]. Because footwear conditions were not considered in data extraction, their potential influence could not be assessed. This limitation does not apply directly to measurements of strength, power and muscle mass performed with objective methods. Moreover, only two studies reported the reliability of asymmetry indices, showing considerable differences between measurements [15,36]. None of the included studies assessed the incremental predictive value of asymmetry by comparing models with and without it. It therefore cannot be concluded whether asymmetry improves fall prediction beyond established functional indicators.

GRADE ratings should be interpreted as a measure of the certainty of evidence relating to a specific exposure–outcome pair. The very low certainty of evidence found for all pairs assessed means that the results of future, appropriately designed studies may change both the estimate of the association analyzed and its interpretation [25]. Consistency in the direction of the observed effects alone does not eliminate the limitations resulting from the cross-sectional nature of most of the data. In turn, the inability to formally assess funnel plot asymmetry means that publication bias can neither be confirmed nor ruled out. The more frequent occurrence of positive results in studies with small sample sizes is therefore a reason for cautious interpretation of the body of evidence, but it is not in itself sufficient evidence of selective publication. Several mechanisms may explain why the largest studies at the lowest risk of bias more often reported null or inverse findings, whereas positive findings predominated in small case–control and cross-sectional studies. Small studies are more susceptible to publication bias and to selective reporting of significant results among multiple indices; case–control comparisons of selected fallers and non-fallers may inflate the strength of associations through spectrum bias and differential recall bias; and retrospective fall ascertainment allows for reverse causation. At the same time, the large studies addressed specific constructs, so their findings cannot be generalized to other domains. The published literature may therefore overrepresent positive associations, and isolated positive findings from small studies analyzing multiple indices should be regarded as hypothesis-generating.

### 4.6. Limitations of the Study

Not extracting values from graphs limited the range of available estimates. Moreover, the potential overlap between the samples of James et al. [19,26] means that these reports should not be treated as fully independent replications.

A limitation is the use of our own summary thresholds for the NOS and AXIS [27,28]. They facilitate the description of the evidence, but total scores may obscure problems within individual domains, and the results of the two tools should not be compared directly. Because these thresholds are not a feature of the instruments themselves, studies scoring close to a boundary could be classified differently under alternative conventions. For this reason, the summary categories were used descriptively, and GRADE judgements were based on limitations at the level of individual items. Specific limitations, such as the lack of control for muscle capacity or the retrospective ascertainment of falls, are of greater interpretive importance. In addition, the predominance of community-dwelling individuals and the exclusion of populations defined by a unilateral pathology limit the transferability of the conclusions to institutional care. The findings do not apply to populations with neurological conditions or post-injury populations, such as people with post-stroke hemiparesis or unilateral fracture, in whom asymmetry is a direct consequence of the lesion.

Clinical trial registers and grey literature were not searched, because observational studies are rarely registered.

### 4.7. Practical Implications

Two potential uses of the lower-limb asymmetry parameter should be distinguished. As a complementary parameter, interpreted within an assessment together with bilateral strength, power or muscle mass and functional tests, asymmetry may help to describe an individual’s functional profile. As an independent screening or diagnostic criterion for fall risk, its use is not supported by current evidence. None of the included studies demonstrated incremental predictive value beyond established measures, and no universal threshold of asymmetry considered pathological can be derived from the available data.

The results do not justify the use of a single asymmetry index or a single percentage threshold as a standalone tool for assessing the risk of falls. If asymmetry is included in the assessment, its interpretation should go beyond the relative index alone and include the values obtained for both limbs and the specific nature of the task performed. Low asymmetry occurring together with bilaterally reduced performance cannot be treated as equivalent to normal function, just as a greater difference between the limbs does not in itself constitute evidence of performance problems. The observed associations concerning gait speed and selected loading parameters indicate that further assessment of the potential complementary value of asymmetry measurements is warranted; however, current data do not justify using them as a substitute for direct functional assessment [14,16,17,20,21].

In the broader context of fall prevention, there is considerably stronger evidence for exercise than for the normalization of asymmetry. The Cochrane review by Sherrington et al. [51] included 108 randomized trials; in the meta-analysis of the rate of falls, exercise reduced the rate by 23% compared with control (rate ratio 0.77; 95% CI 0.71–0.83; 59 trials, 12,981 participants; high-certainty evidence). Benefits were shown for balance and functional exercises, among other types. Comparison with this review indicates that the rationale for implementing exercise is stronger than the rationale for setting a specific level of symmetry as a therapeutic goal. It is not known whether an improvement in asymmetry mediates the reduction in falls; the effectiveness of exercise programs should not be presented as evidence of such a mechanism.

This review did not include intervention studies and does not allow us to determine whether a reduction in asymmetry decreases the incidence of falls or improves mobility. A change in the index alone need not signify a benefit either: it may result from an improvement in the weaker limb, a deterioration in the stronger limb or simultaneous changes on both sides. Accordingly, interventions specifically targeting asymmetry cannot be recommended on the basis of this review; whether reducing asymmetry improves patient outcomes requires evidence from interventional studies.

### 4.8. Future Directions

Future research should focus on addressing the methodological limitations and gaps identified in this review. To this end, the following research directions are proposed. Attention should be paid to conducting prospective studies including women and men with diverse levels of physical performance, with prospective recording of falls, e.g., using monthly calendars. Sample size planning should take into account the expected number of events, whereas first and recurrent falls should be analyzed as separate outcomes. In future work, authors should standardize the methods of measuring and reporting asymmetry as far as possible. A more advantageous approach would be to present the absolute values of parameters for both limbs alongside the relative asymmetry indices. It is necessary to report the calculation formulas and the measurement reliability parameters determined in the target population.

The incremental prognostic value of asymmetry should be determined in accordance with the recommendations for prognostic factor research [52]. This requires analyses comparing models that include asymmetry with models without this parameter, while simultaneously controlling for the functional parameters of both limbs. Model evaluation should include discriminative ability. Cut-off points derived from exploratory analyses should undergo independent external validation before being used in clinical practice.

Future studies should also include standardization of measurement conditions in studies of gait asymmetry and lower-limb load distribution, taking into account the type of footwear. These factors should be documented to increase the comparability of results across studies.

Once the independent prognostic value of asymmetry has been confirmed in subsequent studies, it will be justified to conduct randomized controlled trials assessing the effectiveness of interventions targeting the weaker limb. Their aim should be to establish whether such an approach provides greater clinical benefits than conventional exercise programs. Changes in asymmetry should be analyzed in conjunction with changes in the parameters of both limbs, treating them as complementary indicators of the response to the intervention.

## 5. Conclusions

Lower-limb asymmetry is not a homogeneous construct, and its associations with falls and physical performance in individuals aged 60 years and older depend on the measurement domain and the outcome assessed. The available evidence does not confirm universal and independent prognostic value of strength and power asymmetry. Greater muscle mass asymmetry shows a more consistent association with lower gait speed, whereas selected measures of gait and loading asymmetry provide preliminary prognostic evidence from two small cohorts. These findings remain limited by the observational nature of the studies and the very low certainty of evidence in all comparisons assessed. They apply to the general older population.

At present, no universal threshold of pathological asymmetry can be identified, nor can it be stated that its reduction prevents falls. Asymmetry measurements should be interpreted in relation to bilateral muscle capacity and the demands of the task. Further studies should establish which indices provide reproducible information beyond existing performance measures and whether their modification leads to benefits that are relevant to older adults.

## Data Availability

The original contributions presented in the study are included in the article; further inquiries can be directed to the corresponding author.

## Supplementary Materials

The following supporting information can be downloaded at: https://www.mdpi.com/article/10.3390/jcm15208198/s1, Table S1: PRISMA 2020 checklist.

## Appendix A. Full Search Strategies

### Appendix A.1. Scopus

TITLE-ABS-KEY (“older adult*” OR “older people” OR “older person*” OR “older men” OR “older women” OR elder* OR geriatric* OR senior* OR aging OR ageing OR “old age” OR “community dwelling” OR “community-dwelling” OR “nursing home*” OR septuagenarian* OR octogenarian* OR nonagenarian*) AND TITLE-ABS-KEY (asymmetr* OR symmetr* OR interlimb OR “inter-limb” OR “limb symmetry index” OR “symmetry index” OR “symmetry angle” OR “asymmetry index” OR “bilateral deficit” OR “side to side difference*” OR “between limb difference*” OR “limb dominance” OR “leg dominance” OR “stronger limb” OR “weaker limb” OR “dominant leg” OR “nondominant leg” OR “non-dominant leg” OR imbalance) AND TITLE-ABS-KEY (“lower limb*” OR “lower extremit*” OR leg OR legs OR thigh* OR knee* OR hip OR hips OR ankle* OR quadricep* OR hamstring* OR calf OR “plantar flexor*” OR “knee extensor*” OR “hip abductor*” OR gait OR walking OR “step length” OR stride OR “stance phase” OR “swing phase” OR “ground reaction force*” OR “single leg” OR “one leg” OR unipedal OR “lean mass” OR “muscle mass” OR “muscle strength” OR “cross-sectional area”) AND TITLE-ABS-KEY (fall OR falls OR falling OR faller* OR fallen OR balance OR “postural control” OR “postural stability” OR “postural sway” OR “center of pressure” OR “centre of pressure” OR “gait speed” OR “walking speed” OR mobility OR “physical function*” OR “physical performance” OR “functional capacity” OR “functional performance” OR “timed up and go” OR TUG OR “short physical performance battery” OR SPPB OR “sit to stand” OR “chair stand” OR “chair rise” OR “Berg balance” OR “Mini-BESTest” OR BESTest OR Tinetti OR “six minute walk” OR “6 minute walk” OR frailty OR frail OR sarcopeni* OR “fear of falling” OR “falls efficacy” OR “balance confidence” OR disabilit* ) AND NOT DOCTYPE (re OR ch OR le OR ed OR no OR cp OR sh)

### Appendix A.2. Web of Science

TS=(“older adult*” OR “older people” OR “older person*” OR “older men” OR “older women” OR elder* OR geriatric* OR senior* OR aging OR ageing OR “old age” OR “community dwelling” OR “nursing home*” OR septuagenarian* OR octogenarian* OR nonagenarian*) AND TS=(asymmetr* OR symmetr* OR interlimb OR “inter-limb” OR “limb symmetry index” OR “symmetry index” OR “symmetry angle” OR “asymmetry index” OR “bilateral deficit” OR “side to side difference*” OR “between limb difference*” OR “limb dominance” OR “leg dominance” OR “stronger limb” OR “weaker limb” OR “dominant leg” OR “non-dominant leg” OR imbalance) AND TS=(“lower limb*” OR “lower extremit*” OR leg OR legs OR thigh* OR knee* OR hip OR hips OR ankle* OR quadricep* OR hamstring* OR calf OR “plantar flexor*” OR “knee extensor*” OR “hip abductor*” OR gait OR walking OR “step length” OR stride OR “stance phase” OR “swing phase” OR “ground reaction force*” OR “single leg” OR “one leg” OR unipedal OR “lean mass” OR “muscle mass” OR “muscle strength” OR “cross-sectional area”) AND TS=(fall OR falls OR falling OR faller* OR fallen OR balance OR “postural control” OR “postural stability” OR “postural sway” OR “center of pressure” OR “centre of pressure” OR “gait speed” OR “walking speed” OR mobility OR “physical function*” OR “physical performance” OR “functional capacity” OR “functional performance” OR “timed up and go” OR TUG OR “short physical performance battery” OR SPPB OR “sit to stand” OR “chair stand” OR “chair rise” OR “Berg balance” OR “Mini-BESTest” OR BESTest OR Tinetti OR “six minute walk” OR “6 minute walk” OR frailty OR frail OR sarcopeni* OR “fear of falling” OR “falls efficacy” OR “balance confidence” OR disabilit*)

### Appendix A.3. PubMed

(“older adult*”[tw] OR “older people”[tw] OR “older person*”[tw] OR “older men”[tw] OR “older women”[tw] OR elder*[tw] OR geriatric*[tw] OR senior*[tw] OR aging[tw] OR ageing[tw] OR “old age”[tw] OR “community dwelling”[tw] OR “community-dwelling”[tw] OR “nursing home*”[tw] OR septuagenarian*[tw] OR octogenarian*[tw] OR nonagenarian*[tw] OR “Aged”[Mesh] OR “Aged, 80 and over”[Mesh] OR “Geriatrics”[Mesh] OR “Independent Living”[Mesh] OR “Homes for the Aged”[Mesh]) AND (asymmetr*[tw] OR symmetr*[tw] OR interlimb[tw] OR “inter-limb”[tw] OR “limb symmetry index”[tw] OR “symmetry index”[tw] OR “symmetry angle”[tw] OR “asymmetry index”[tw] OR “bilateral deficit”[tw] OR “side to side difference*”[tw] OR “side-to-side difference*”[tw] OR “between limb difference*”[tw] OR “between-limb difference*”[tw] OR “limb dominance”[tw] OR “leg dominance”[tw] OR “stronger limb”[tw] OR “weaker limb”[tw] OR “dominant leg”[tw] OR “nondominant leg”[tw] OR “non-dominant leg”[tw] OR imbalance[tw] OR “Functional Laterality”[Mesh]) AND (“lower limb*”[tw] OR “lower extremit*”[tw] OR leg[tw] OR legs[tw] OR thigh*[tw] OR knee*[tw] OR hip[tw] OR hips[tw] OR ankle*[tw] OR quadricep*[tw] OR hamstring*[tw] OR calf[tw] OR “plantar flexor*”[tw] OR “knee extensor*”[tw] OR “hip abductor*”[tw] OR gait[tw] OR walking[tw] OR “step length”[tw] OR stride[tw] OR “stance phase”[tw] OR “swing phase”[tw] OR “ground reaction force*”[tw] OR “single leg”[tw] OR “one leg”[tw] OR unipedal[tw] OR “lean mass”[tw] OR “muscle mass”[tw] OR “muscle strength”[tw] OR “cross-sectional area”[tw] OR “Lower Extremity”[Mesh] OR “Gait”[Mesh] OR “Muscle Strength”[Mesh] OR “Body Composition”[Mesh]) AND (fall[tw] OR falls[tw] OR falling[tw] OR faller*[tw] OR fallen[tw] OR balance[tw] OR “postural control”[tw] OR “postural stability”[tw] OR “postural sway”[tw] OR “center of pressure”[tw] OR “centre of pressure”[tw] OR “gait speed”[tw] OR “walking speed”[tw] OR mobility[tw] OR “physical function*”[tw] OR “physical performance”[tw] OR “functional capacity”[tw] OR “functional performance”[tw] OR “timed up and go”[tw] OR TUG[tw] OR “short physical performance battery”[tw] OR SPPB[tw] OR “sit to stand”[tw] OR “chair stand”[tw] OR “chair rise”[tw] OR “Berg balance”[tw] OR “Mini-BESTest”[tw] OR BESTest[tw] OR Tinetti[tw] OR “six minute walk”[tw] OR “6 minute walk”[tw] OR frailty[tw] OR frail[tw] OR sarcopeni*[tw] OR “fear of falling”[tw] OR “falls efficacy”[tw] OR “balance confidence”[tw] OR disabilit*[tw] OR “Accidental Falls”[Mesh] OR “Postural Balance”[Mesh] OR “Frailty”[Mesh] OR “Sarcopenia”[Mesh] OR “Mobility Limitation”[Mesh] OR “Physical Functional Performance”[Mesh])

### Appendix A.4. EBSCOhost

(TI (“older adult*” OR “older people” OR “older person*” OR elderly OR geriatric* OR aging OR ageing OR “old age” OR octogenarian* OR nonagenarian*) OR AB (“older adult*” OR “older people” OR “older person*” OR elderly OR geriatric* OR “community dwelling” OR “nursing home*” OR “aged 60” OR “aged 65” OR “60 years and older” OR “65 years and older” OR octogenarian* OR nonagenarian*)) AND (TI ((asymmetr* OR symmetr* OR “side-to-side” OR interlimb OR “inter-limb” OR “between-limb”) N5 (limb* OR leg* OR lower OR gait OR step* OR stride* OR strength OR power OR muscle* OR knee* OR hip* OR ankle* OR mass OR force*)) OR AB ((asymmetr* OR symmetr* OR “side-to-side” OR interlimb OR “inter-limb” OR “between-limb”) N5 (limb* OR leg* OR lower OR gait OR step* OR stride* OR strength OR power OR muscle* OR knee* OR hip* OR ankle* OR mass OR force*)) OR TI (“limb symmetry index” OR “symmetry index” OR “asymmetry index” OR “symmetry angle” OR “gait asymmetry” OR “bilateral deficit” OR “stronger limb” OR “weaker limb” OR “limb dominance” OR “leg dominance”) OR AB (“limb symmetry index” OR “symmetry index” OR “asymmetry index” OR “symmetry angle” OR “gait asymmetry” OR “bilateral deficit” OR “stronger limb” OR “weaker limb” OR “limb dominance” OR “leg dominance”)) AND (TI (fall OR falls OR falling OR faller* OR “postural control” OR “postural stability” OR “postural sway” OR “gait speed” OR “walking speed” OR “physical function*” OR “physical performance” OR “functional capacity” OR “timed up and go” OR TUG OR SPPB OR “sit to stand” OR “chair stand” OR “Berg balance” OR BESTest OR Tinetti OR “six minute walk” OR sarcopeni* OR frailty ) OR AB (fall OR falls OR falling OR faller* OR “fear of falling” OR “falls efficacy” OR “postural control” OR “postural stability” OR “postural sway” OR “gait speed” OR “walking speed” OR “physical function*” OR “physical performance” OR “functional capacity” OR “timed up and go” OR TUG OR “short physical performance battery” OR SPPB OR “sit to stand” OR “chair stand” OR “Berg balance” OR “Mini-BESTest” OR Tinetti OR “six minute walk” OR sarcopeni* OR frailty)) NOT (PT (review OR “systematic review” OR “meta analysis” OR editorial OR comment OR letter OR news OR “book review”) OR DT (review OR editorial OR “book review”) OR TI (review OR “meta-analys*” OR “scoping review” OR “narrative review” OR bibliometric*) OR AB (“this systematic review” OR “PRISMA guidelines” OR PROSPERO)) NOT (TI (stroke OR hemipare* OR amputat* OR Parkinson* OR “cerebral palsy” OR “multiple sclerosis” OR “anterior cruciate” OR arthroplast* OR athlet* OR soccer OR football OR children OR adolescen* OR pediatric OR paediatric))
